# Antioxidant evaluation and computational prediction of prospective drug-like compounds from polyphenolic-rich extract of *Hibiscus cannabinus* L. seed as antidiabetic and neuroprotective targets: assessment through in vitro and in silico studies

**DOI:** 10.1186/s12906-023-04023-7

**Published:** 2023-06-19

**Authors:** Olakunle Bamikole Afolabi, Oluwaseun Ruth Olasehinde, Damilola Grace Olanipon, Samson Olatunde Mabayoje, Olufemi Michael Familua, Kikelomo Folake Jaiyesimi, Esther Kemi Agboola, Tolulope Olajumoke Idowu, Olabisi Tajudeen Obafemi, Oyindamola Adeniyi Olaoye, Omotade Ibidun Oloyede

**Affiliations:** 1 Phytomedicine and Toxicology Unit, Biochemistry Programme, Department of Chemical Sciences, College of Sciences, Afe-Babalola University, P.M.B 5454, Ado-Ekiti, Ekiti State Nigeria; 2 Department of Medical Biochemistry, College of Medicine and Health Sciences, Afe Babalola University, P.M.B 5454, Ado-Ekiti, Ekiti State Nigeria; 3 Department of Biological Sciences, College of Sciences, Afe Babalola University, P.M.B. 5454, Ado-Ekiti, Ekiti State Nigeria; 4 Department of Pharmacology and Toxicology, College of Pharmacy, Afe Babalola University, P.M.B. 5454, Ado-Ekiti, Ekiti State Nigeria; 5 Medicinal Plant Unit, Chemistry Programme, Department of Chemical Sciences, College of Sciences, Afe-Babalola University, P.M.B 5454, Ado- Ekiti, Ekiti State Nigeria; 6 Department of Biochemistry, Ekiti State University, P.M.B 5363, Ado-Ekiti, Ekiti State Nigeria

**Keywords:** *Hibiscus cannabinus* seed, Antidiabetic agent, Neurodegenerative conditions, MM-GBSA scores, Molecular docking

## Abstract

**Background:**

Reports have implicated diabetes mellitus (DM) and Alzheimer’s disease (AD) as some of the global persistent health challenges with no lasting solutions, despite of significant inputs of modern-day pharmaceutical firms. This study therefore, aimed to appraise the in vitro antioxidant potential, enzymes inhibitory activities, and as well carry out in silico study on bioactive compounds from polyphenolic-rich extract of *Hibiscus cannabinus* seed (PEHc).

**Methods:**

In vitro antioxidant assays were performed on PEHc using standard methods while the identification of phytoconstituents was carried out with high performance liquid chromatography (HPLC). For the in silico molecular docking using Schrodinger’s Grid-based ligand docking with energetics software, seven target proteins were retrieved from the database (https://www.rcsb.org/).

**Results:**

HPLC technique identified twelve chemical compounds in PEHc, while antioxidant quantification revealed higher total phenolic contents (243.5 ± 0.71 mg GAE/g) than total flavonoid contents (54.06 ± 0.09 mg QE/g) with a significant (*p* < 0.05) inhibition of ABTS (IC_50_ = 218.30 ± 0.87 µg/ml) and 1, 1-diphenyl-2-picrylhydrazyl free radicals (IC_50_ = 227.79 ± 0.74 µg/ml). In a similar manner, the extract demonstrated a significant (*p* < 0.05) inhibitory activity against α-amylase (IC_50_ = 256.88 ± 6.15 µg/ml) and α-glucosidase (IC_50_ = 183.19 ± 0.23 µg/ml) as well as acetylcholinesterase (IC_50_ = 262.95 ± 1.47 µg/ml) and butyrylcholinesterase (IC_50_ = 189.97 ± 0.82 µg/ml), respectively. Furthermore, In silico study showed that hibiscetin (a lead) revealed a very strong binding affinity energies for DPP-4, (PDB ID: 1RWQ) and α-amylase (PDB ID: 1SMD), gamma-tocopherol ( for peptide-1 receptor; PDB ID: 3C59, AChE; PDB ID: 4EY7 and BChE; PDB ID: 7B04), cianidanol for α-glucosidase; PDB ID: 7KBJ and kaempferol for Poly [ADP-ribose] polymerase 1 (PARP-1); PDB ID: 6BHV, respectively. More so, ADMET scores revealed drug-like potentials of the lead compounds identified in PEHc.

**Conclusion:**

As a result, the findings of this study point to potential drug-able compounds in PEHc that could be useful for the management of DM and AD.

**Supplementary Information:**

The online version contains supplementary material available at 10.1186/s12906-023-04023-7.

## Introduction

The importance of bioactive plant secondary metabolites in the management of a variety of human diseases, including cancer, diabetes mellitus (DM), cognitive dysfunction or memory impairment, such as Alzheimer’s disease (AD), Parkinson’s disease, and others, has recently been highlighted [1, 2]. DM and AD are now recognized as two of the most common and fatal health issues among the elderly [3]. Increasing evidence suggests that oxidative stress (redox imbalance) plays critical roles in the pathophysiogenesis of a wide range of human diseases [4]. oxidative stress occurs when the endogenous antioxidant system is overburdened by the proliferation of reactive oxygen species (ROS)/electrophiles [5]. These reactive electrophiles disrupt the cellular architectures and functions of human vital tissues such as the pancreas, kidneys, brain, and liver [6].

DM is a chronic metabolic disease that is one of the most serious public health issues of the twenty-first century [7]. It is a diverse group of disorders characterized by hyperglycemia caused by improper glucose metabolism [8]. DM has been identified as a major contributor to the rising global mortality rate [9]. According to recent statistics, DM affects approximately 4 per cent of the global population and is expected to rise to 5.4 percent by 2025 [10]. Diabetes-related chronic hyperglycemia causes protein glycation, which leads to a number of secondary complications, including retinopathy, nephropathy, neuropathy, and atherosclerotic vascular disease [11, 12].

DM is classified into two types: type 1 (T1DM) and type 2 (T2DM) [13]. T2DM is thought to account for up to 95% of the diabetic population [14]. T2DM is characterized by hyperglycemia caused by insufficient insulin from the pancreatic -cell and insulin resistance [15]. Recent studies have linked proliferated ROS to changes in the insulinotropic activity of pancreatic -cells in the etiology of type I and type II diabetes [16, 17]. However, one of the most recent approaches to treating postprandial hyperglycemia in T2DM is to delay the metabolism of dietary carbohydrates by inhibiting alpha-glucosidase and alpha-amylase enzymatic activities [18]. These two key enzymes are involved in starch breakdown and glucose absorption in the intestine [19]. Alpha amylase and alpha glucosidase inhibitors are two potential targets for the development of lead compounds for the treatment of T2DM. Nevertheless, a number of synthetic drugs, such as oral hypoglycemic drugs (e.g., metformin, sulfonylureas, thiazolidinediones, biguanides, meglitinides, and dipeptidyl peptidase-IV (DPP-4) inhibitors), have been developed over time [20, 21].

Similarly, AD is a progressive neurodegenerative brain disorder [22–24]. It is the most common cause of dementia, especially in the elderly, and affects nearly 44 million people worldwide [25]. It is frequently characterized by a loss of cholinergic neuron structure and function. The brain’s vulnerability due to low antioxidant capacity has been identified as a significant damaging factor in AD pathogenesis [26]. Redox imbalance impairs neural functions and irreversibly destroys cellular macromolecules [27]. Agents that restore acetylcholine (ACh) levels by inhibiting major forms of cholinesterases (i.e., acetylcholinesterase, AChE and butyrylcholinesterase, BChE) have been reported to improve cholinergic transmission in neuronal tissue in the treatment of AD [28]. ACh is a neurotransmitter that modulates memory function in both normal and neurodegenerative conditions. AChE and BChE are important regulators of the ACh and cholinergic signaling systems [29]. These two enzymes are found in the brain and are extremely efficient, cleaving over 10,000 ACh molecules released into the synaptic cleft per second. The hydrolytic actions of these cholinesterases disrupt the cholinergic system levels [30, 31]. There are currently a number of potential inhibitors of these Ach-specific hydrolytic enzymes available, including donepezil, galantamine, physostigmine, rivastigmine, and tacrine [32]. However, these inhibitors have been associated with some side effects and are only effective against mild forms of AD, and there are currently no drugs that inhibit BChE activity [33]. As a result, new drugs are needed to combat AD.

Nowadays, there are more and more new therapeutic targets available for drug discovery as a result of the completion of the human genome project [34]. These developments enable computational methodologies to pervade all aspects of drug discovery today [35, 36], including virtual screening (VS) methods for lead optimization and hit detection [37]. A prominent computational technique for structure-based lead identification is similar to high throughput screening (HTS), in that compounds from a virtual database are screened for their expected affinity to a certain protein target [38]. The docked protein-ligand geometries reveal information about the binding process’s driving factors [39]. Protein-ligand complexes play critical roles in biological systems. Ligands define protein function by acting as a substrate, inhibitor, activator, cofactor, signal inducer, and allosteric regulator. The structure of these complexes helps to analyze interactions between proteins and their ligands, allowing for a better understanding of the molecular basis of their function [40]. In investigating such interactions, the delineation of hydrogen bonds produced by the protein with its bound ligands is critical. There are currently embryonic evidences that support the prominent roles of plant-based secondary metabolites in the screening for the lead targets and dependable inhibitors in averting the onset and delaying the progression of neurodegenerative diseases and other prevailing human illnesses [41].

*Hibiscus cannabinus* (*H. cannabinus*) seed, commonly known as kenaf, is an annual herbaceous crop, which belongs to the *Malvaceae* family that probably originated from sub-Saharan Africa [42, 43]. It is considered as an important fiber crop with numerous industrial applications [44]. Phytochemical analysis of *H. cannabinus* has revealed the presence of various bioactive compounds, including flavonoids, phenolic acids, tannins, alkaloids, and saponins [45]. These metabolites have been shown to possess antioxidant and anti-inflammatory properties, which may contribute to the its medicinal properties. Also, several studies have investigated the antioxidant activity of *H. cannabinus* extracts. One study of Gupta et al. [46], found that methanol and aqueous extracts of *H. cannabinus* had strong antioxidant activities, as evidenced by their ability to scavenge free radicals and inhibit lipid peroxidation. More so, documented study has indicated hypoglycemic activity and insulin sensitivity enhancing effect of solvent extracts of the plant [47]. Currently, the plant is still being explored for its medicinal properties. Therefore, this study was designed to investigate in vitro antioxidant potentials, enzymes inhibitory activities (i.e., α-glucosidase, α-amylase, AChE and BChE inhibitions) and in silico docking of the HPLC-identified lead compounds of polyphenolic-rich extract of *H. cannabinus* (PEHc) against some protein targets that are crucial in T2DM and neurodegenerative disease (AD).

## Materials and methods

### Materials

#### Chemicals and reagents used

Intestinal α-glucosidase, pancreatic α-amylase, *p*-nitrophenyl-α-D-glucopyranose (PNPG), 1, 1-diphenyl-2-picrylhydrazyl (DPPH), acetylcholine iodide, butyrylcholine iodide, acarbose, Ellman’s reagent (5,5’-dithiobis (2-nitrobenzoic acid), DTNB), 2,2- azinobis (3-ethyl-benzothiazoline-6-sulfonic acid) (ABTS), butylated hydroxytoluene (BHT), gallic acid, quercetin and were procured from Sigma-Aldrich, Inc., (Saint Louis, MO), and other chemicals used were of analytical grades and prepared in all-glass apparatus using sterilized distilled water.

### Collection of samples and preparation

#### Collection of sample

The seeds of H. cannabinus were purchased in a single transaction from a vendor in Ado-Ekiti, Ekiti State, Nigeria. Mr Omotayo, a taxonomist, authenticated the sample (not a wild variety) at the Plant Science and Biotechnology Department of Ekiti State University (Ado-Ekiti, Ekiti State, Nigeria). The sample was air-dried for 14 days before being pulverized with a laboratory blender and stored at room temperature until further use.

#### Preparation of polyphenolic-rich extract

The extraction of polyphenolic-rich extract of *H. cannabinus* seed (PEHc) was carried out as described by the Chu et al. [48]. The blended sample (10 g) was extracted with 80% acetone (1:5 w/v) for 2 h with stirring periodically (every 5 min interval) at room temperature. The mixture was filtered (using muslin cloth) and filtrate evaporated using waterbath at 45℃ to dryness. The dried phenolic-rich extract was stored at -8℃ in the refrigerator until further bioassays.

### Methods

#### In vitro phytochemical and antioxidant assays

##### Determination of total phenolic content (TPC)

The TPC of PEHc was determined using the method of Singleton et al. [49]. The extract (0.2 ml) was mixed with 2.5 ml 10% Folin–Ciocalteu reagent and 2 ml 7.5% sodium carbonate. The reaction mixture was subsequently incubated at 45 °C for 40 min, the absorbance was measured at 700 nm with garlic acid as standard, and the result expressed in microgram gallic acid equivalent/gram dried sample (μg GAE/g dry sample).

##### Determination of total flavonoid content (TFC)

The TFC of PEHc was determined using a colorimetric assay described by Bao et al. [50]. The extract (0.2 ml) was added to 0.3 ml 5% NaNO_3_ at zero time. After 5 min, 0.6 ml 10% AlCl_3_ was added and after 6 min, 2 ml 1 M NaOH was added to the mixture followed by the addition of 2.1 ml distilled water. Absorbance was read at 510 nm against the reagent blank and flavonoid content was expressed as microgram quercetin equivalent/ gram dry sample (μg QE/g dry sample).

##### DPPH free radical scavenging ability of PEHc

The DPPH free radical inhibitory ability of PEHc was performed according to the method described by Gyamfi et al. [51]. Briefly, appropriate dilution of the extract in different concentrations with distilled water (1 ml) was mixed with an equal volume (1 ml) of DPPH in methanol. The mixture was kept in the dark for 30 min and absorbance levels were taken at wavelength 516 nm in the spectrophotometer. The DPPH free radical inhibitory ability was thereafter calculated and expressed as % inhibition against the control.

##### ABTS free radical scavenging assay

ABTS free radical scavenging ability of PEHc was carried out using the methods of Zhao et al. [52], with some modifications. The sample (0.2 ml) in various concentrations was mixed with 2.0 ml diluted ABTS radical cation solution (7 mM ABTS dissolved in 0.01 M PBS, pH 7.4). The reaction mixture was left at room temperature for 20 min, absorbance was measured immediately at 734 nm in the spectrophotometer. ABTS free radical scavenging ability was subsequently calculated and expressed as % inhibition against the control with BHT as standard.

#### Enzyme inhibitory activity of PEHc

##### Pancreatic α-amylase inhibition assay

The α-amylase inhibitory activity of PEHc was determined according to the method described by Shai et al. [53], with slight modifications based on the spectrophotometric assay. A volume of (0–250 μl) PEHc was incubated with 500 μl of porcine pancreatic amylase (2 U/ml) in 0.1 M phosphate buffer (pH 6.8) at 37 °C for 20 min. One percent soluble starch (200 μl) dissolved in 0.1 M phosphate buffer (pH 6.8) was then added to the reaction mixture and incubated at 37 °C for 1 h. DNSA (1 ml) was thereafter added and boiled for 10 min. The absorbance of the resulting mixtures were measured at 540 nm and the inhibitory activity was calculated and expressed as % inhibition against the control.

##### Intestinal α-glucosidase inhibition assay

The *α*-glucosidase inhibitory activity of PEHc was determined according to the method described by Ademiluyi and Oboh [54], with slight modifications. Briefly, 0–100 μl of the extract was incubated with 100 μl α-glucosidase (1.0 U/ml) solution in phosphate buffer (0.1 M, pH 6.8) at 37 °C for 15 min. Thereafter, 50 μl pNPG solution (5 mM) in phosphate buffer (0.1 M, pH 6.8) was added and the mixture was further incubated at 37 °C for 20 min. The absorbance of the released *p*-nitrophenol was measured at 405 nm and the inhibitory activity was calculated and expressed as % inhibition against the control.

##### Acetylcholinesterase and butyrylcholinesterase inhibition assay

Cholinesterases (AChE and BChE) inhibition assays were assessed using a modified colorimetric method of Ellman as described by Perry et al. [55]. The AChE and BChE activities were determined in a reaction mixture with total volume of 1 ml, containing phosphate buffer (0.1 M, pH 8.0), DTNB (10 mM), 0.05 ml cytosol and acetylcholine iodide/ butyrylcholine iodide (150 mM). Change in absorbance was monitored at 412 nm by UV spectrophotometer for 180 s at 25 °C. The AChE or BChE inhibitory activity was thereafter calculated and expressed as % inhibition against the control.

#### Determination of IC_50_

The PEHc concentration required to cause 50% inhibition (IC_50_) was calculated using a linear regression curve generated from a plot of the percentage inhibition caused by the extracts versus different concentrations (µg/ml) of the extract used [56].

#### Quantification of phytochemicals using HPLC coupled with diode array detector

Chromatographic analysis was performed using uBandapak C18 reversed‐phase column (250 mm × 4.6 mm) packed with 5‐μm diameter particles; the mobile phase was water with 1% formic acid (v/v) (solvent A) and HPLC grade methanol (solvent B) at a flow rate of 0.6 mL/min and injection volume 50 μL. This mobile phase was filtered through a 0.45‐μm membrane filter (Millipore), then deaerated ultrasonically prior to use. Appropriate detection wavelengths were used for detection of different compounds in the PEHc. The chromatographic peaks of the analytes were thereafter confirmed by comparing their retention time (Rt) and UV spectra with those of the reference standards. All chromatographic operations were carried out at ambient temperature following the analytical protocol by Afolabi et al. [57].

#### In silico and molecular docking studies

##### Preparation of protein targets

The X-ray crystal structure of DPP-4 (PDB ID: 1RWQ), α-amylase (PDB ID: 1SMD), glucagon-like peptide-1 receptor (PDB ID: 3C59), α-glucosidase (PDB ID: 7KBJ), Poly [ADP-ribose] polymerase 1 (PARP-1) (PDB ID: 6BHV), acetylcholinesterase (AChE)-(PDB ID: 4EY7), and butyrylcholinesterase (BChE)-(PDB ID: 7B04) were obtained from protein data bank (https://www.rcsb.org/) and further prepared using Glide’s protein preparation wizard [58].

##### Preparation of ligands

All compounds obtained from HPLC analysis (P-hydroxybenzoic acid, gallic acid, gamma-tocophenol, caffeic acid, beta-sitosterol, catechin, vanillic acid, syringic acid, hibiscetine, kaempferol, ferulic acid, and linalool) and standard drugs for individual targets (obtained from medexpress and drugbank) were prepared using the LigPrep 2.4 software [59]. The OPLS-2005 force field was employed for optimization, which resulted in the ligand’s low-energy conformer [60].

##### Molecular docking

To evaluate the docking parameters, all drugs were docked into the DPP-4, (PDB ID: 1RWQ), α-amylase (PDB ID: 1SMD), glucagon-like peptide-1 receptor (PDB ID: 3C59), α-glucosidase (PDB ID: 7KBJ), poly [ADP-ribose] polymerase 1 (PARP-1) (PDB ID: 6BHV), acetylcholinesterase (AChE)-(PDB ID: 4EY7), and butyrylcholinesterase (BChE)-(PDB ID: 7B04) proteins retrieved from the protein database, using Schrodinger’s Grid-Based Ligand Docking with Energetics (GLIDE) software. In the receptor grid generation with a partial atomic charge of 0.25, the scaling factor for protein van der Waals radii was 1.0 to soften the potential for nonpolar regions of the receptors. The GLIDE docking parameters were set to the flexible potential function’s default. For all docking studies, no limitations or constraints were applied. GLIDE 5.6’s receptor grid generation module was used to define the active site for docking ligands. Co-crystallied ligands were chosen, and grids were generated around the active site of 1RWQ, 1SMD, 3C59, 7KBJ, 6BHV, 4EY7 and 7B04 with receptors van der Waals scale of 0.9 for non-polar atoms. The active sites were determined by a radius of 10 around the crystal structure’s ligands [61, 62].

##### Prime MM-GBSA calculations

The prime molecular mechanistic generalized born surface area (MM-GBSA) technique was used to compute the free energy of binding. The simulations were run in Prime, version 2.2, with the generalized born surface area (GBSA) continuum model [63, 64]. The binding free energy (ΔG_binding_), was computed using the method of Lyne et al. [65], as shown in Eq. (1).

1 $$\Delta {\mathrm{G}}_{\mathrm{binding }}=\mathrm{\Delta E}+\Delta {\mathrm{G}}_{\mathrm{solvation}}+\Delta {\mathrm{G}}_{\mathrm{SA}}$$

2 $$\mathrm{\Delta E}={\mathrm{E}}_{\mathrm{complex}}-{\mathrm{E}}_{\mathrm{protein}}-{\mathrm{E}}_{\mathrm{ligand}}$$

Where in (1 & 2) ΔG_binding_, binding free energy; ΔG_SA,_ free energy of surface area; ΔG_solvation_^.^ solvation free energy (1); ΔE, free minimized energy; E_complex_, E_protein_, and E_ligand_ are the minimized energies of the protein–inhibitor complex, protein and inhibitor, respectively.

3 $$\Delta {\mathrm{G}}_{\mathrm{solvation}}={\mathrm{G}}_{\mathrm{solvation\, }(\mathrm{complex})}-{\mathrm{G}}_{\mathrm{solvation\, }(\mathrm{protein})}-{\mathrm{G}}_{\mathrm{solvation\, }(\mathrm{ligand})}$$

Where in (3); G_solvation (complex)_, G_solvation (protein)_, and G_solvation (ligand)_ are the solvation free energies of the complex, protein, and inhibitor (ligand), respectively:

4 $$\Delta {\mathrm{G}}_{\mathrm{SA}}={\mathrm{G}}_{\mathrm{SA\, }(\mathrm{complex})}-{\mathrm{G}}_{\mathrm{SA\, }(\mathrm{protein})}-{\mathrm{G}}_{\mathrm{SA\, }(\mathrm{ligand})}$$

Where in (4); G_SA (complex)_, G_SA (protein)_, and G_SA (ligand)_ are the complex, protein, and inhibitor surface area energies, respectively.

### Data analyses

Data were analyzed using GraphPad Prism 8.0 (Version 8, Software Program, GraphPad Prism Inc., San Diego, CA). Results were presented as mean ± SD. One-way analysis of variance (ANOVA) was used for the analyses of biochemical indices, followed by Tukey’s post-hoc test. Significant differences were considered at *p* < 0.05.

## Results

### Phytochemical and antioxidant capacity of PEHc

**Fig. 1:**
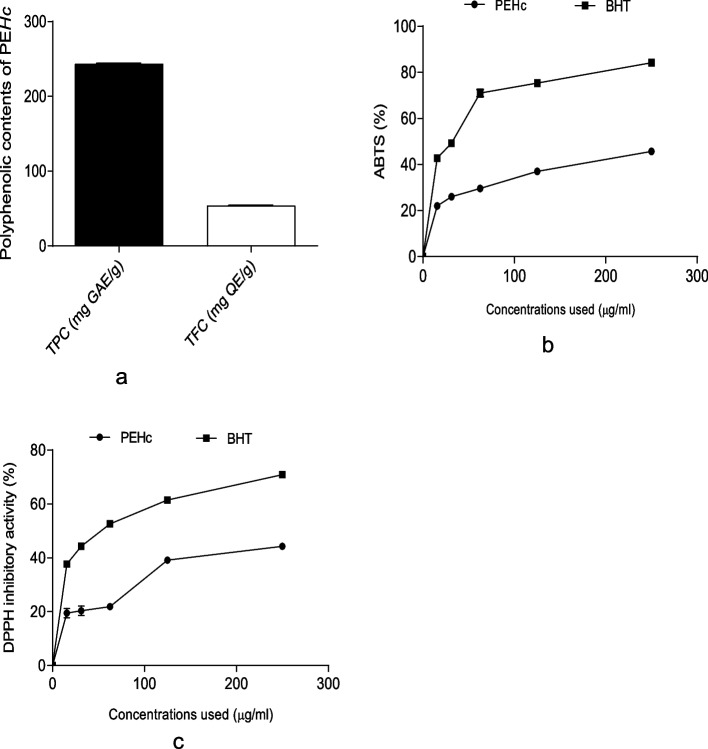
**a** Total phenolic and total flavonoid contents; (**b**) ABTS percentage (%) inhibitory activity and; (**c**) DPPH percentage (%) inhibitory activity of polyphenolic-rich extract of *Hibiscus cannabinus* seed (PEHc). Note: ABTS, 2, 2-azinobis (3-ethylbenzothiazoline-6-sulfonic acid); BHT, Butylated hydroxytoluene; DPPH, 1,1-diphenyl-2-picryl hydrazyl

Figure 1a, b & c indicate the total phenolic and total flavonoid contents, % ABTS inhibitory activity and % DPPH inhibitory activity of PEHc. As shown in Fig. 1a, PEHc revealed higher TPC (243.5 ± 0.71 mg GAE/g) than TFC (54.06 ± 0.09 mg QE/g). Also, in Fig. 1b, PEHc demonstrated a significant (*p* < 0.05) inhibitory activity against ABTS free radical (IC_50_ = 218.30 ± 0.87 µg/ml; Table 1) in a concentration-dependent manner, which was twofold weaker than the standard control; BHT (IC_50_ = 112.15 ± 0.12 µg/ml; Table 1). Similarly, in Fig. 1c, PEHc revealed a significant (*p* < 0.05) inhibitory activity against DPPH free radical (IC_50_ = 227.79 ± 0.74 µg/ml; Table 1) in a concentration-dependent that was weaker compared to the standard control; BHT (IC_50_ = 135.67 ± 0.81 µg/ml; Table 1).

**Table 1:** IC_50_ values (µg/ml) of the polyphenolic-rich extract of *Hibiscus cannabinus* (PEHc) on DPPH, α-amylase, α-glucosidase, ABTS, BchE and AchE % inhibitions

	PEHc	BHT	Acarbose	Galanthamine
DPPH	227.79 ± 0.74	135.67 ± 0.81		
α-Amylase	256.88 ± 6.15		154.51 ± 0.00	
α-Glucosidase	183.19 ± 0.23		121.42 ± 0.13	
ABTS	218.30 ± 0.87	112.15 ± 0.12		
AchE	262.95 ± 1.47			153.92 ± 0.10
BchE	189.97 ± 0.82			134.95 ± 0.16

Results represent mean ± SD of duplicate trials (*n* = 2)

### Enzyme inhibitory activity of PEHc

**Fig. 2:**
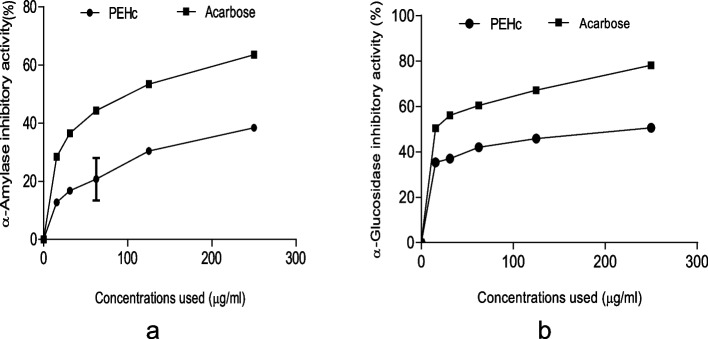
Percentage (%) inhibitory potential of PEHc against (**a**) α-amylase and (**b**) α-glucosidase carbohydrate-hydrolyzing enzymatic activities

Figure 2a & b represent the % inhibitory potential of PEHc against (a) α-amylase and (b) α-glucosidase carbohydrate-hydrolyzing enzymatic activities. As revealed in Fig. 2a, PEHc demonstrated a significant (*p* < 0.05) inhibitory activity against α-amylase with IC_50_ = 256.88 ± 6.15 µg/ml (Table 1), which was weaker compared to a known starch blocker; acarbose with IC_50_ = 154.51 ± 0.00 µg/ml (Table 1). Similarly, in Fig. 2b, PEHc showed a significant (*p* < 0.05) inhibition against the activity of α-glucosidase with IC_50_ = 183.19 ± 0.23 µg/ml (Table 1), which was also weaker compared to a standard control; acarbose with IC_50_ = 121.42 ± 0.13 µg/ml (Table 1).

**Fig. 3:**
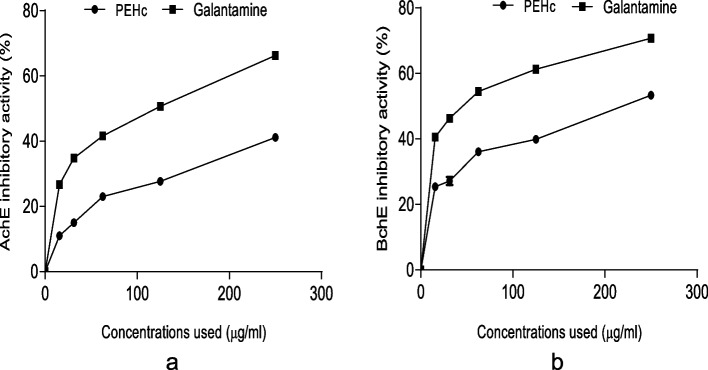
Percentage (%) inhibitory potential of polyphenolic-rich extract of *Hibiscus cannabinus* seed (PEHc) against (**a**) AChE and (**b**) BChE enzymatic activities

Figure 3a & b represent the % inhibitory potential of PEHc against (a) AChE and (b) BChE enzymatic activities. As shown in Fig. 3a, PEHc revealed a significant (*p* < 0.05) inhibitory activity against AChE with IC_50_ = 262.95 ± 1.47 µg/ml (Table 1), which was weaker compared to the activity of a standard control; galantamine with IC_50_ = 153.92 ± 0.10 µg/ml (Table 1). Similarly, as seen in Fig. 3b, PEHc demonstrated a significant (*p* < 0.05) inhibitory activity against BChE with IC_50_ = 189.97 ± 0.82 µg/ml (Table 1), which was also weaker compared to a standard control; galantamine with IC_50_ = 134.95 ± 0.16 µg/ml (Table 1).

### High performance liquid chromatographic analyses

**Fig. 4:**
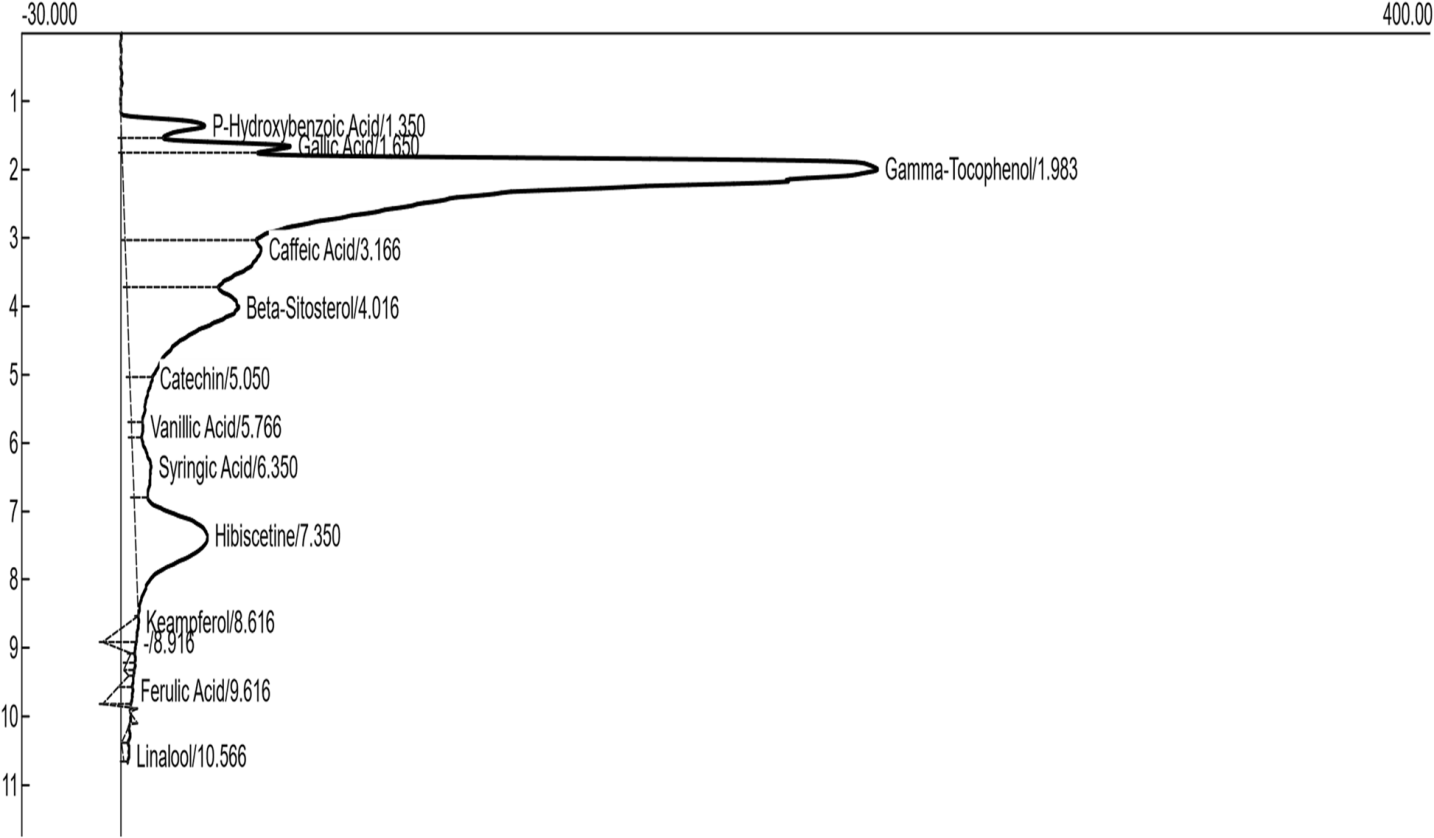
HPLC fingerprinting of phytochemical compositions of polyphenolic-rich extract of *Hibiscus cannabinus* seed (PEHc)

Figure 4 and Table 2 present HPLC fingerprinting and phytochemical compositions of PEHc. As shown in Fig. 4, twelve (12) chemical compounds were identified in PEHc using HPLC, namely; *p*-hydroxybenzoic acid, gallic acid, gamma-tocophenol, caffeic acid, beta-sitosterol, catechin, vanillic acid, syringic acid, hibiscetine, keampferol, ferulic acid, and linalool.

**Table 2:** HPLC analysis of the phytochemical compositions of polyphenolic-rich extract of *Hibiscus cannabinus* (PEHc)

Compounds	Retention time	Peak area	Peak height	Units
P-Hydroxybenzoic Acid	1.35	337.02	25.41	ppm
Gallic Acid	1.65	471.10	51.17	ppm
Gamma-Tocophenol	1.98	9170.30	228.66	ppm
Caffeic Acid	3.17	1503.01	41.16	ppm
Beta-Sitosterol	4.02	1636.51	33.76	ppm
Catechin	5.05	198.10	6.85	ppm
Vanillic Acid	5.77	42.80	3.48	ppm
Syringic Acid	6.35	239.60	5.56	ppm
Hibiscetine	7.35	1061.39	21.87	ppm
Keampferol	8.62	120.84	2.57	ppm
Ferulic Acid	9.62	98.26	5.41	ppm
Linalool	10.57	30.03	1.88	ppm

### In silico and molecular docking analysis

**Fig. 5:**
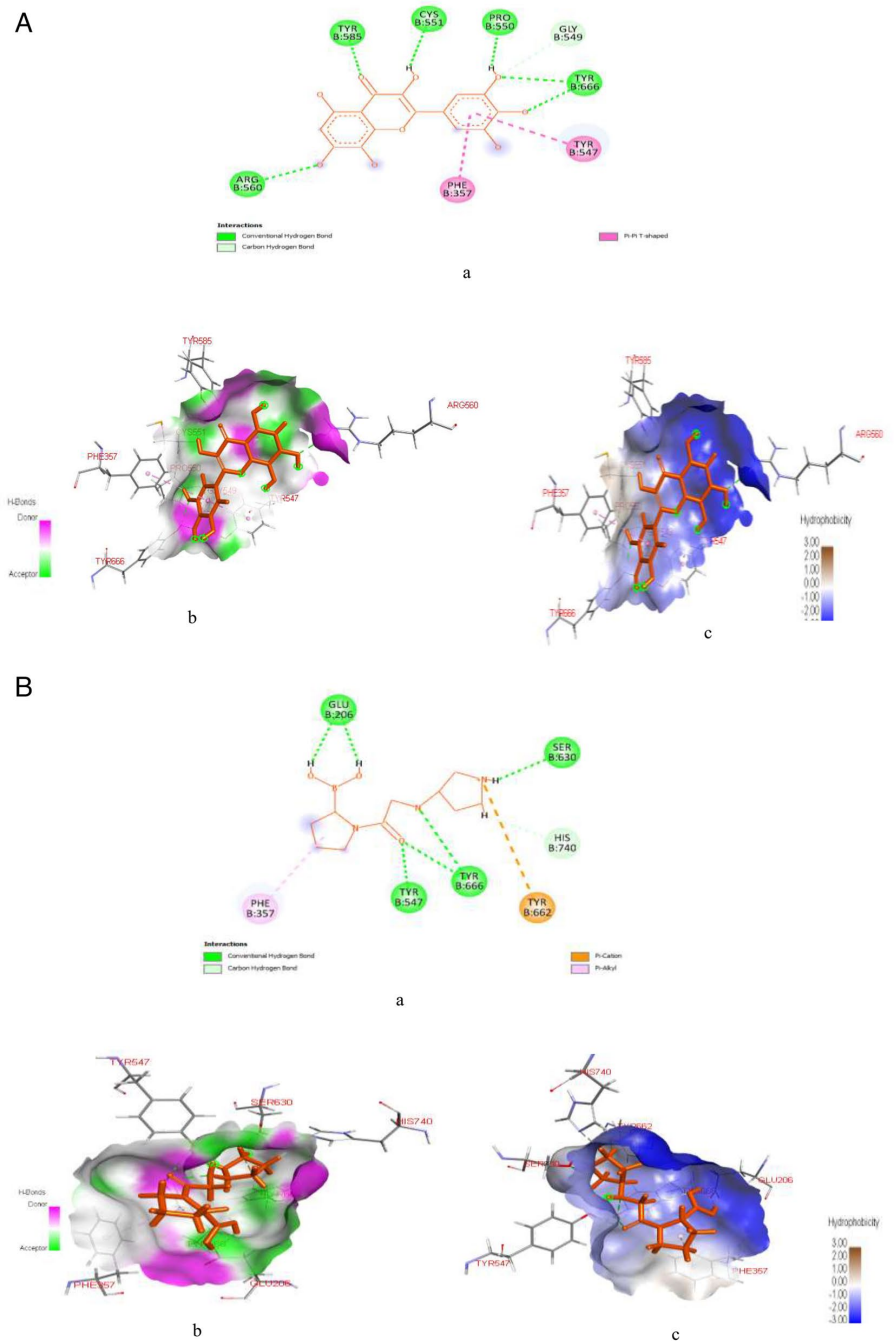
**A**: Post-docking analyses of dipeptidyl peptidase IV protein (1RWQ) target in complex with hibiscetin. **a** 2D representation of the dipeptidyl peptidase IV-hibiscetin complex; (**b**) Hydrogen acceptors and donor interactions around hibiscetin (**c**) Hydrophobic cloud interactions around hibiscetin. **B**: Post-docking analyses of dipeptidyl peptidase IV protein (1RWQ) target in complex with Dutogliptin. **a** 2D representation of the Dipeptidyl peptidase IV-Dutogliptin complex; (**b**) Hydrogen acceptors and donor interactions around Dutogliptin; (**c**) Hydrophobic cloud interactions around Dutogliptin

Figure 5A & B represent post-docking analyses of DPP-4 protein target in complex with hibiscetin (lead compound) and dutogliptin (a known standard DPP-4 inhibitor). The 2D representations of the binding of hibiscetin and dutogliptin to DPP-4 protein targets are shown in Fig. 5Aa & Ba. The complexation of hibiscetin with DPP-4 revealed SP (-7.52 kcal/mol), XPG (-7.559 kcal/mol) and MM-GBSA (-40.22 kcal/mol) binding scores while dutogliptin binding with DPP-4 protein target revealed SP (-9.342 kcal/mol), XPG (-9.351 kcal/mol) and MM-GBSA (-35.04 kcal/mol) binding scores as seen in Table S1. As indicated in the Fig. 5Ab and Bb, interaction of the lead compound with DPP-4 revealed formation of six hydrogen bonds with some amino acids at specific bond distances as; ARG560 (2.467), TYR585 (1.652), TYR666 (2.371), TYR666 (2.087), CYS551 (2.349), PRO550 (1.741), and GLY549 (3.003), whereas, interaction of dutogliptin with the target protein showed seven hydrogen bonds with DPP-4 at TYR547 (1.601), two TYR666 (2.523 and 2.550), two GLU206 (2.146 and 1.827), SER630 (2.191), and HIS740 (2.652). Also, hibiscetin revealed the formation of two hydrophobic interactions with the DPP-4 protein target at PHE357 (5.306) and TYR547 (4.603), both of which are precisely Pi-Pi T-shaped interactions, while dutogliptin demonstrated two hydrophobic interactions with DPP-4 protein at TYR662 (4.619) and PHE357 (4.376), as indicated in Fig. 5Ac and Bc.

**Fig. 6:**
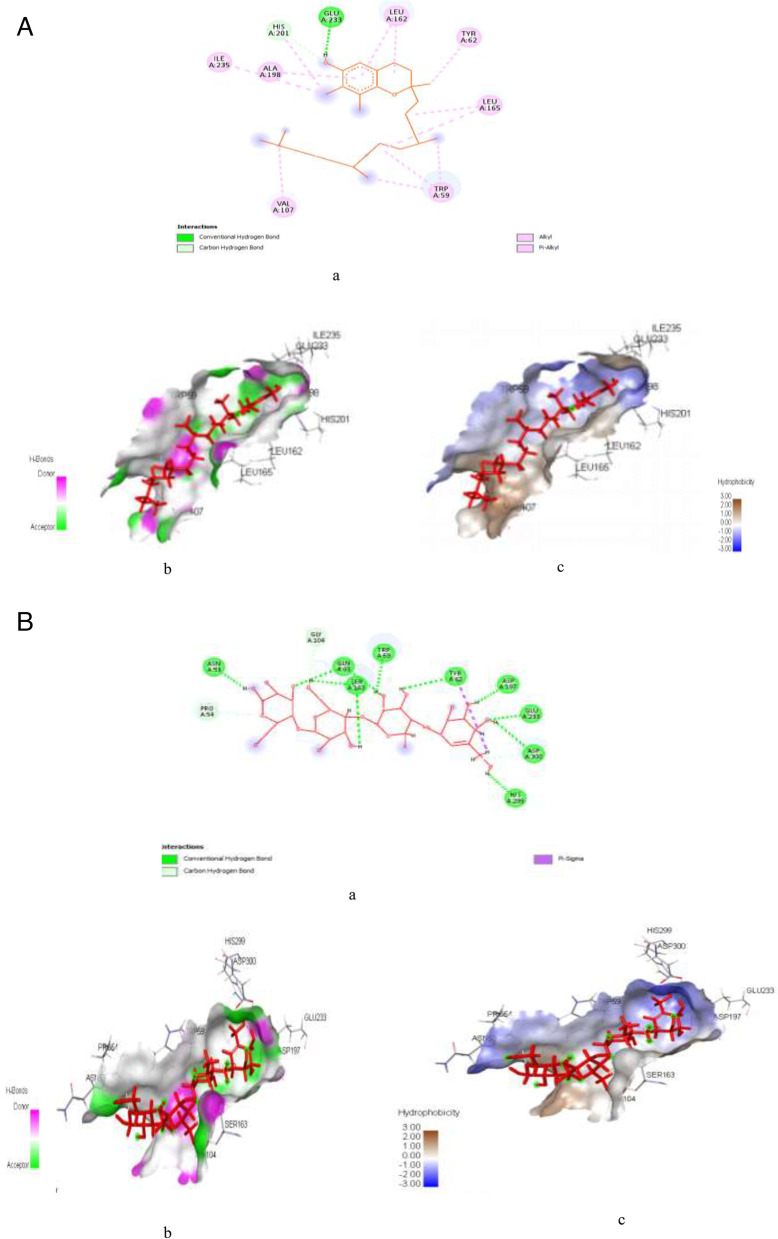
**A**: Post-docking analyses of α-amylase protein (1SMD) in complex with hibiscetin. **a** 2D representation of the alpha amylase- Hibiscetin complex; (**b**) Hydrogen acceptors and donors interactions around hibiscetin; (**c**) Hydrophobic cloud interactions around hibiscetin. **B**: Post-docking analyses of α-amylase protein (1SMD) in complex with acarbose. **a** 2D representation of the alpha amylase- acarbose complex; (**b**) Hydrogen acceptors and donor interactions around acarbose; (**c**) Hydrophobic cloud interactions around acarbose

Figure 6A & B represent post-docking analyses of α-amylase protein in complex with hibiscetin (lead compound) and acarbose. In the result, the 2D representations of the binding of hibiscetin and acarbose with α-amylase protein targets are presented in Fig. 6Aa & Ba. The complexation of hibiscetin with α-amylase revealed SP (-9.303 kcal/mol), XPG (-9.34 kcal/mol) and MM-GBSA (-35.2 kcal/mol) binding scores, while binding of acarbose had SP (-12.011 kcal/mol), XPG (-12.339 kcal/mol) and MM-GBSA (-78.1 kcal/mol) binding scores as seen in Table S2. Also, as indicated in Fig. 6Ab & Bb, the hydrogen interaction of hibiscetin with α-amylase formed two hydrogen bond at GLU233 (1.76344) and HIS201 (2.64579), while acarbose formed eighteen hydrogen bond interactions with α-amylase at GLN63 (1.75871), GLN63 (1.7255), GLU233 (1.76788), TYR62 (2.15108), TRP59 (2.01124), SER163 (2.04107), ASP197 (1.90742), ASP300 (2.70907), SER163 (1.76363), ASN53 (1.76582), HIS299 (1.84284), PRO54 (2.81372), GLY104 (2.75587), SER163 (2.43421), SER163 (2.87948), SER163 (2.63473), ASP197 (2.38009), and ASP300 (2.60679). Similarly, as seen in Fig. 6Ac & Bc, hibiscetin formed thirteen hydrophobic interactions with α-amylase at specific amino acids with their bond distances i.e., LEU162 (4.9379), LEU165 (4.78145), LEU165 (5.26765), VAL107 (4.62826), ILE235 (4.89209), TRP59 (4.23627), TRP59 (5.1283), TRP59 (4.48415), TRP59 (4.76896), TYR62 (4.67719), HIS201 (5.0987), LEU162 (4.55897), and ALA198 (5.07087), whereas acarbose revealed one hydrophobic interactions with α-amylase protein target at TYR62 (2.5496).

**Fig. 7:**
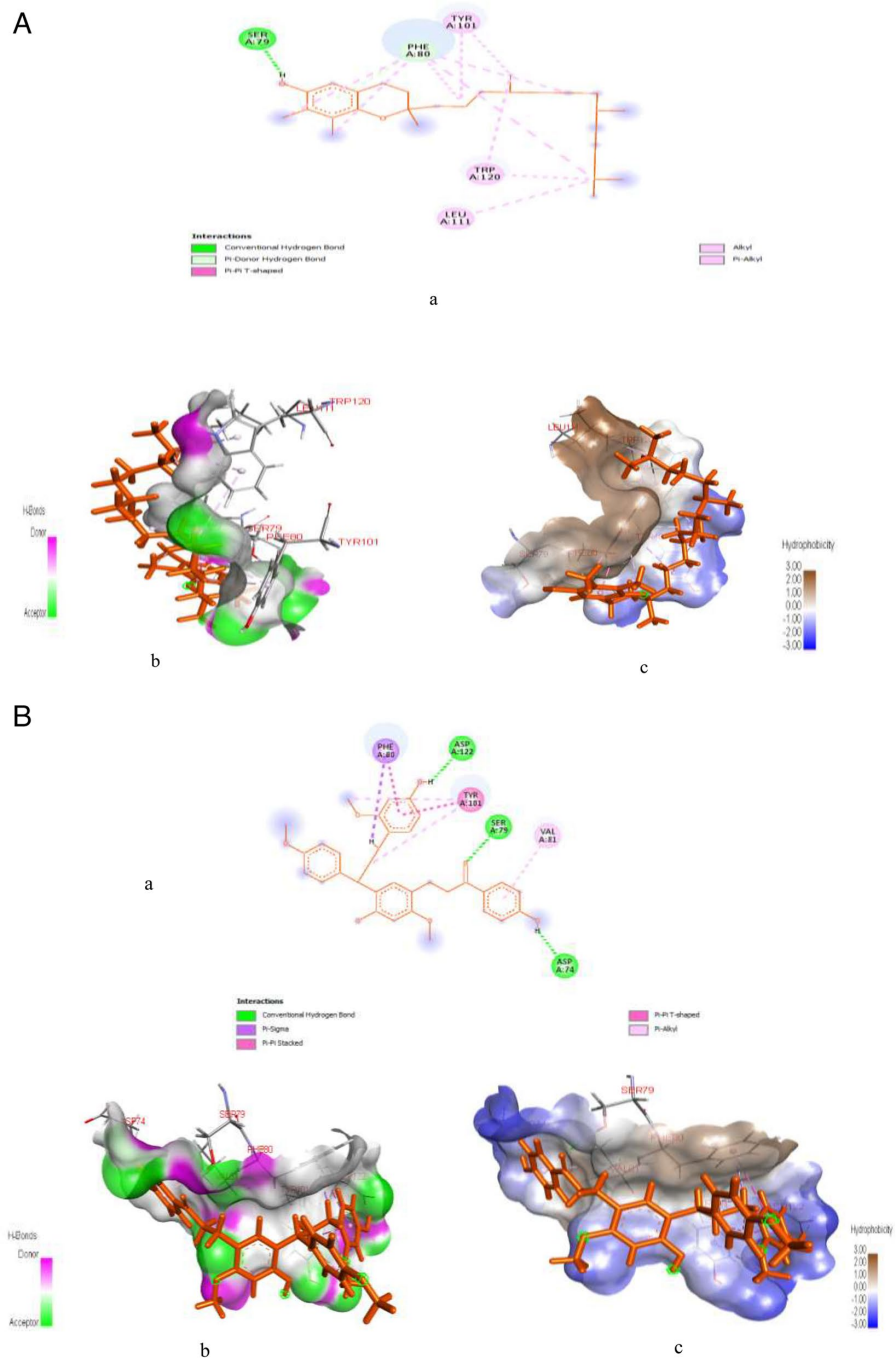
**A**: Post-docking analyses of glucagon-like peptide-1 receptor protein (3C59) in complex with gamma-Tocopherol. **a** 2D representation of the glucagon-like peptide-1 receptor- gamma Tocopherol complex; (**b**) Hydrogen acceptors and donor interactions around gamma-Tocopherol; (**c**) Hydrophobic cloud interactions around gamma-Tocopherol. **B**: Post-docking analyses of glucagon-like peptide-1 receptor protein (3C59) in complex with cochinchinenin C (**a**) 2D representation of the glucagon-like peptide-1 receptor protein; (**b**) Hydrogen acceptors and donor interactions around cochinchinenin **C**; (**c**) Hydrophobic cloud interactions around cochinchinenin C

Figure 7A & B represents post-docking analyses of glucagon-like peptide-1 receptor in complex with gamma-tocopherol (lead compound) and cochinchinenin C. In the result, the 2D representations of the glucagon-like peptide-1 receptor interactions with gamma-tocopherol and cochinchinenin C are presented in Fig. 7Aa & Ba. The binding of gamma-tocopherol with glucagon-like peptide-1 receptor as seen in Table S3, revealed SP (-3.137), XPG (-3.137) and MM-GBSA (-46.36) scores while cochinchinenin C interaction with glucagon-like peptide-1 receptor had SP (-2.631), XPG (-2.637) and MM-GBSA (-53.43) scores as shown in Table S3. Also, from the Fig. 7Ab & Bb, gamma-tocopherol forms two hydrogen bond interactions with glucagon-like peptide-1 receptor at SER79 (1.6939) and PHE80 (3.08515), while cochinchinenin C forms three hydrogen bonds interactions at SER79 (1.86323), ASP122 (1.72624), and ASP74 (1.71031). As shown in Fig. 7Ac, gamma-tocopherol interaction with glucagon-like peptide-1 receptor indicated the formation of twelve (12) hydrophobic bond at PHE80 (5.17396), LEU111 (4.9235), PHE80 (5.23651), PHE80 (4.14779), PHE80 (5.43703), PHE80 (4.87961), PHE80 (5.38966), TYR101 (3.8441), TYR101 (4.17908), TRP120 (4.20415), TRP120 (5.49086), and TRP120 (4.40369) with all these hydrophobic bonds involving in Pi-alkyl interactions except PHE80 (5.17396) which formed Pi-Pi T-shaped interaction, whereas, cochinchinenin C interaction with glucagon-like peptide-1 receptor, as seen in Fig. 7Bc reveals six hydrophobic interactions at PHE80 (2.6722), TYR101 (3.73311), PHE80 (5.64996), TYR101 (4.64707), TYR101 (4.95487) and VAL81 (4.18158) with PHE80 (2.6722), TYR101 (3.73311) and PHE80 (5.64996) involving in Pi-sigma, Pi-Pi stacked, and Pi-Pi T-shaped hydrophobic bonds interactions and TYR101 (4.64707), TYR101 (4.95487) and VAL81 (4.18158) involving in Pi-alkyl interactions.

**Fig. 8:**
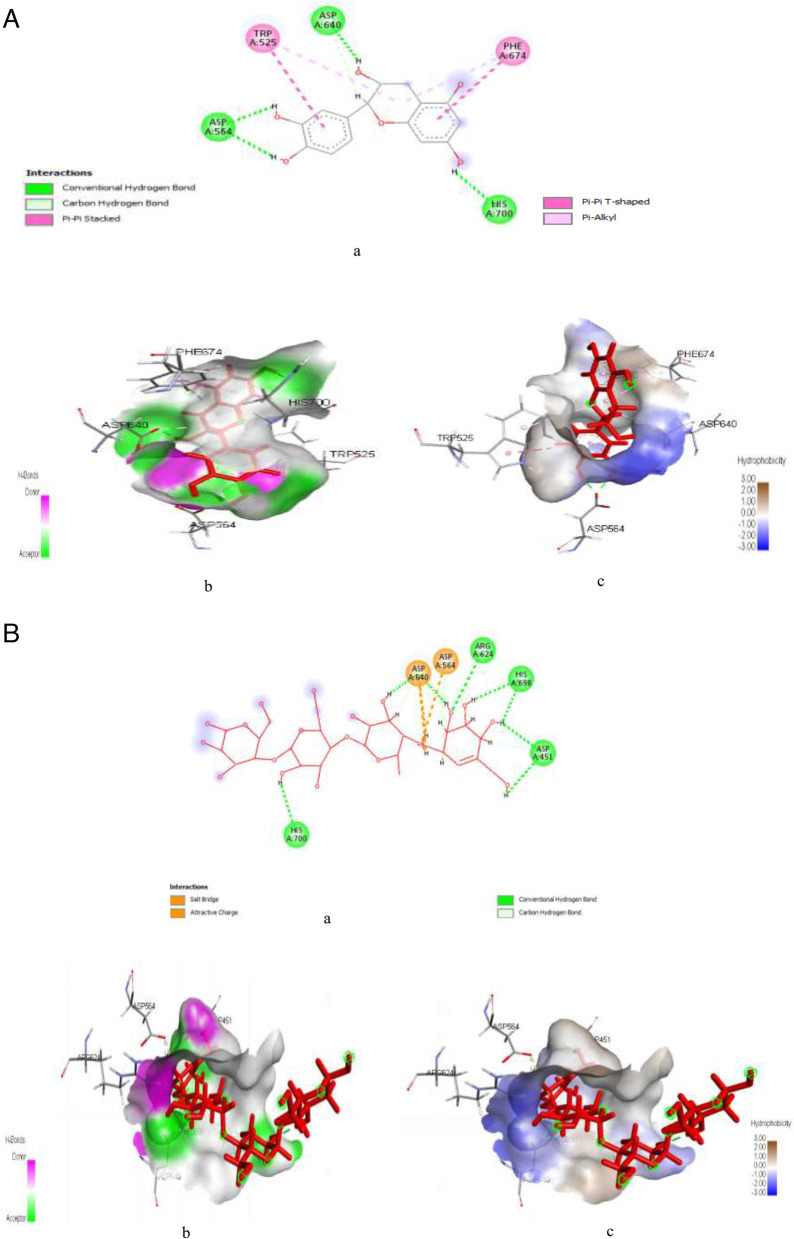
**A**: Post-docking analyses of α-glucosidase receptor protein (7KBJ) in complex with cianidanol. **a** 2D representation of the alpha glucosidase receptor protein- cianidanol complex; (**b**) Hydrogen acceptors and donor interactions around cianidanol; (**c**) Hydrophobic cloud interactions around cianidanol. B: Post-docking analyses of α-glucosidase receptor protein (7KBJ) in complex with acarbose. **a** 2D representation of the alpha glucosidase receptor protein- acarbose complex; (**b**) Hydrogen acceptors and donor interactions around acarbose; (**c**) Hydrophobic cloud interactions around acarbose

Figure 8A & B represent post-docking analyses of α-glucosidase receptor protein (PDB ID: 7KBJ) in complex with cianidanol and acarbose. In the result, the 2D representations of α-glucosidase receptor protein interactions with cianidanol and acarbose are presented in Fig. 8Aa & Ba. The binding of α-glucosidase receptor protein this lead compound revealed SP (-7.486), XPG (-7.486) and MM-GBSA (-33.67) scores while acarbose had SP (-11.068), XPG (-11.396) and MM-GBSA (-51.51) scores as seen in Table S4. Also as indicated in the Fig. 8Ab & Bb, binding of α-glucosidase to cianidano formed five hydrogen bonds at ASP640 (1.82655), HIS700 (1.94468), ASP564 (1.70668), ASP564 (1.86806), and ASP640 (2.58708), while interaction with acarbose formed fifteen hydrogen bond at ASP640 (2.14635), ASP640 (2.34973), ARG624 (2.22876), ASP640 (1.92057), HIS700 (1.95912), ASP640 (1.76667), HIS698 (1.94144), ASP451 (1.81677), HIS698 (2.94928), ASP451 (1.81476), ASP640 (2.62922), ASP564 (2.66901), ASP564 (2.39819), ASP640 (2.91182), and ASP451 (3.08489), respectively. Similarly, cianidano interaction with α-glucosidase indicated four hydrophobic interactions at TRP525 (5.99853), PHE674 (4.95415), TRP525 (5.48423), and PHE674 (5.32497) as seen in Fig. 8Ac. However, the amino acid interaction of the protein, i.e., LEU650 (4.87304) with cianidano specifically formed two Pi-Alkyl bonds with TRP525 (5.48423) and PHE674 (5.32497), one Pi-Pi Stacked with TRP525 (5.99853), and one Pi-Pi T-shaped hydrophobic bonds with PHE674 (4.95415). On the other hand, acarbose formed fifteen hydrogen bond interactions with the target protein at ASP640 (2.14635), ASP640 (2.34973), ARG624 (2.22876), ASP640 (1.92057), HIS700 (1.95912), ASP640 (1.76667), HIS698 (1.94144), ASP451 (1.81677), HIS698 (2.94928), ASP451 (1.81476), ASP640 (2.62922), ASP564 (2.66901), ASP564 (2.39819), ASP640 (2.91182) and ASP451 (3.08489) as well as one electrostatic bond at ASP564 (4.61233).

**Fig. 9:**
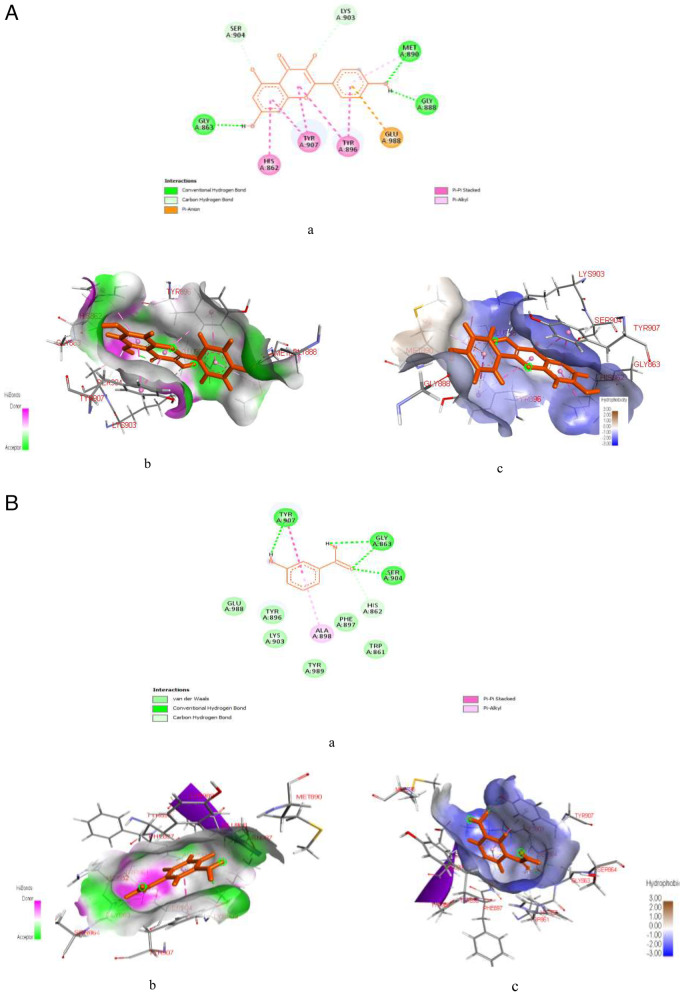
**A**: Post-docking analyses of poly [ADP-ribose] polymerase 1 protein (6BHV) in complex with kaempferol **a**) 2D representation of the Poly [ADP-ribose] polymerase 1- kaempferol complex; (**b**) Hydrogen acceptors and donor interactions around kaempferol; (**c**) Hydrophobic cloud interactions around kaempferol. B: Post-docking analyses of poly [ADP-ribose] polymerase 1 protein (6BHV) in complex with 3-Aminobenzamide. **a** 2D representation of the alpha amylase-3-aminobenzamide complex; (**b**) Hydrogen acceptors and donor interactions around 3-aminobenzamide; (**c**) Hydrophobic cloud interactions around 3-aminobenzamide

Figure 9A & B represent post-docking analyses of poly [ADP-ribose] polymerase 1 in complex with kaempferol (lead compound) and 3-aminobenzamide (reference drug). The 2D representations of poly [ADP-ribose] polymerase 1 interaction with kaempferol and 3-aminobenzamide are indicated in Fig. 9Aa & Ba. The binding of kaempferol with poly [ADP-ribose] polymerase 1 showed a better SP (-9.046 kcal/mol), XPG (-9.078 kcal/mol) and MM-GBSA (-61.69 kcal/mol) binding scores than 3-aminobenzamide with the protein target had SP (-6.646 kcal/mol), XPG (-6.646 kcal/mol) and MM-GBSA (-35.39 kcal/mol) binding scores as shown in Table S5. Also, as indicated in Fig. 9Ab & Bb, interaction of kaempferol with the protein target revealed six hydrogen bonds at MET890 (1.90936), GLY863 (1.69014), GLY888 (1.67182), LYS903 (3.04352), LYS903 (2.68613), SER904 (2.80593), and one electrostatic bond at GLU988 (4.28374), while 3-aminobenzamide interaction with the protein target revealed the formation of three hydrogen bonds at ARG411 (1.83714), ARG411 (1.83825), and SER676 (1.9444). Similarly, as seen in Fig. 9Ac & Bc kaempferol hydrophobic interaction with poly [ADP-ribose] polymerase 1 showed formation of six hydrophobic bonds at HIS862 (4.6465), TYR896 (5.00302), TYR896 (4.31852), TYR907 (3.96858), TYR907 (3.79909) and MET890 (5.20956), while 3-aminobenzamide hydrophobic interaction showed formation of four hydrophobic bonds at LEU650 (4.87304), TRP376 (5.40307), PHE649 (4.80247), and LEU678 (5.12722).

**Fig. 10:**
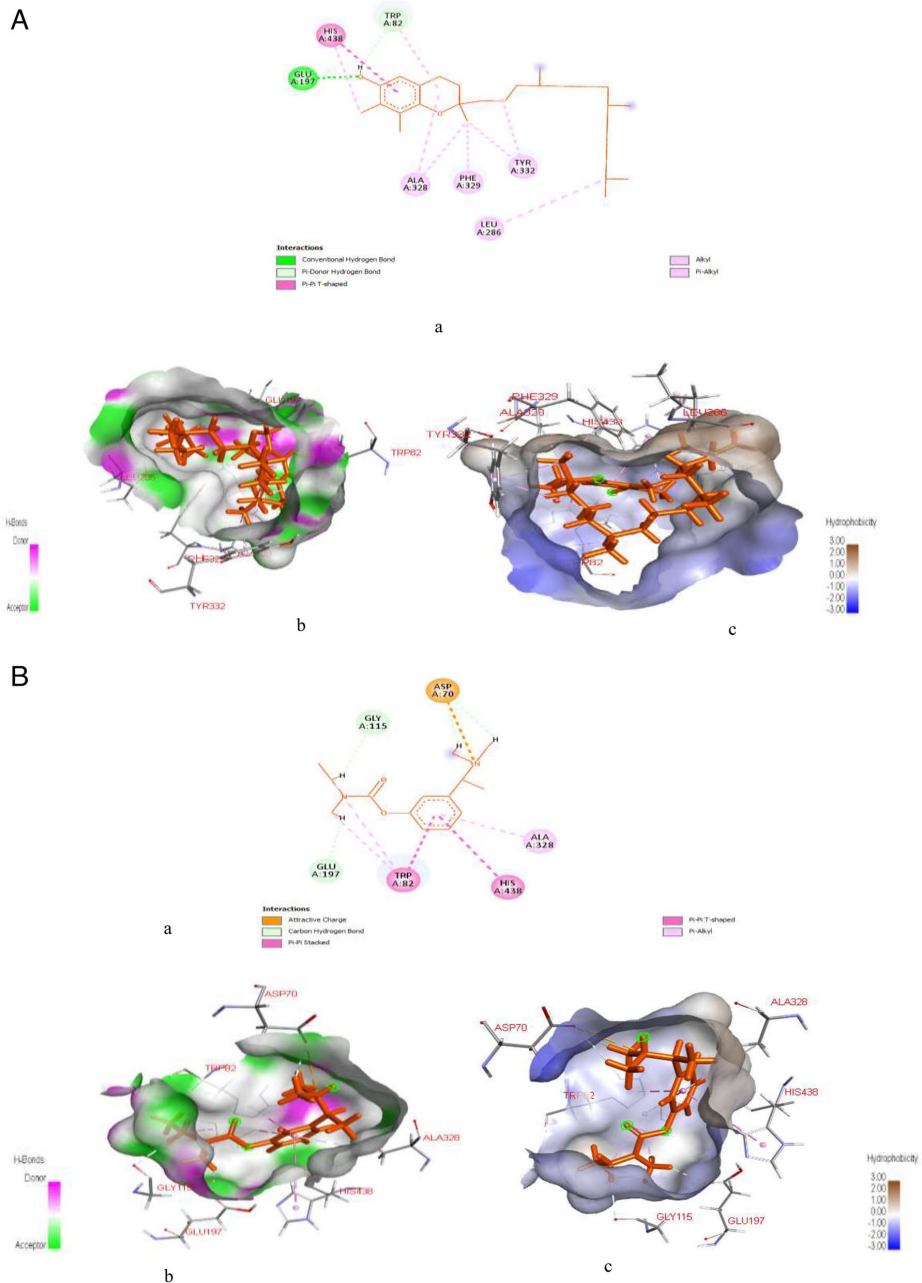
**A**: Post-docking analyses of butyrylcholinesterase protein (7B04) in complex with gamma-Tocopherol. **a** 2D representation of the butyrylcholinesterase protein- gamma tocopherol complex; (**b**) Hydrogen acceptors and donor interactions around gamma-tocopherol; (**c**) Hydrophobic cloud interactions around gamma-Tocopherol. **B**: Post-docking analyses of butyrylcholinesterase protein (7B04) in complex with rivastigmine. **a** 2D representation of the butyrylcholinesterase protein-rivastigmine complex; (**b**) Hydrogen acceptors and donor interactions around rivastigmine; (**c**) Hydrophobic cloud interactions around rivastigmine

Figure 10A & B represent post-docking analyses of butyrylcholinesterase protein in complex with gamma-tocopherol (lead) and rivastigmine (reference drug). The 2D representations of the binding of gamma-tocopherol (lead) and rivastigmine to butyrylcholinesterase protein are shown in Fig. 10Aa & Ba. Also, the binding of gamma-tocopherol with butyrylcholinesterase protein showed SP (-7.716 kcal/mol), XPG (-7.716 kcal/mol) and MM-GBSA (-49.97 kcal/mol) binding scores while rivastigmine had SP (-6.644 kcal/mol), XPG (-6.693 kcal/mol) and MM-GBSA (-49.00 kcal/mol) binding scores as seen in Table S6. However, binding affinity of gamma-tocopherol as the lead compound compete favourable with the reference and other compounds identified in PEHc. Also, as shown in Fig. 10Ab & Bb, hydrogen interaction of gamma-tocopherol with butyrylcholinesterase protein indicated formation of two hydrogen bond at GLU197 (1.78585) and TRP82 (2.41256), whereas rivastigmine interaction showed formation of four hydrogen bond at ASP70 (2.63372), ASP70 (2.71168), GLY115 (2.46635), GLU197 (2.7204), and one electrostatic bond at ASP70 (3.81425). Similarly, as shown in Fig. 10Ac, hydrophobic interactions of gamma-tocopherol with butyrylcholinesterase protein revealed formation of nine hydrophobic bonds at HIS438 (4.80192), ALA328 (4.85732), ALA328 (3.63545), LEU286 (5.4688), TRP82 (4.7633), PHE329 (3.9829), TYR332 (4.70481), TYR332 (4.60073), and HIS438 (4.97202). Out of the nine hydrophobic interaction bond formed, five are precisely Pi-alkyl, i.e., TRP82 (4.7633), PHE329 (3.9829), TYR332 (4.70481), TYR332 (4.60073), and HIS438 (4.97202), while three are alkyl (ALA328 (4.85732), ALA328 (3.63545), LEU286 (5.4688), and HIS438 (4.80192) is Pi-Pi T-shaped. On the other hand, as indicated in Fig. 10Bc, hydrophobic interactions of rivastigmine with butyrylcholinesterase protein showed formation of six hydrophobic bonds at TRP82 (4.10051), TRP82 (4.77074), HIS438 (5.44711), TRP82 (5.42426), TRP82 (4.55835), ALA328 (5.00477). However, the hydrophobic bonds precisely are three Pi-alkyl, i.e., TRP82 (5.42426), TRP82 (4.55835), and ALA328 (5.00477), two Pi-Pi Stacked, i.e., TRP82 (4.10051) and TRP82 (4.77074), and one Pi-Pi T-shaped, i.e., HIS438 (5.44711).

**Fig. 11:**
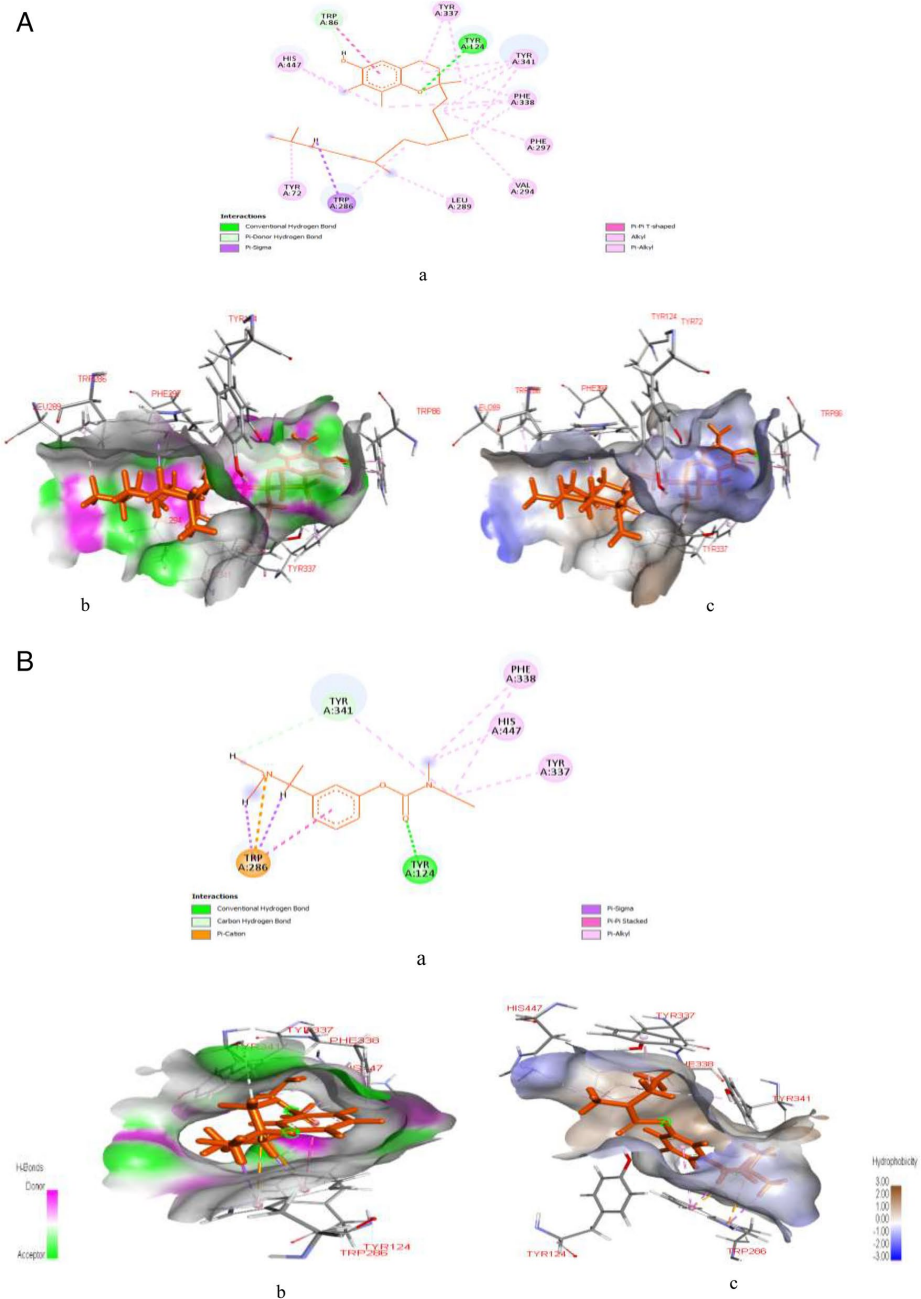
**A**: Post-docking analyses of acetylcholinesterase protein (4EY7) in complex with gamma-Tocopherol. **a** 2D representation of the acetylcholinesterase protein-gamma Tocopherol complex; (**b**) Hydrogen acceptors and donor interactions around gamma-Tocopherol; (**c**) Hydrophobic cloud interactions around gamma-Tocopherol. **B**: Post-docking analyses of acetylcholinesterase protein (4EY7) in complex with rivastigmine. **a** 2D representation of the acetylcholinesterase protein- rivastigmine complex; (**b**) Hydrogen acceptors and donor interactions around rivastigmine; (**c**) Hydrophobic cloud interactions around rivastigmine

Figure 11A & B represents post-docking analyses of acetylcholinesterase protein in complex with gamma-tocopherol and rivastigmine. The 2D representations of the binding of gamma-tocopherol and rivastigmine to acetylcholinesterase protein are shown in Fig. 11Aa & Ba. In addition, the binding of gamma-tocopherol with acetylcholinesterase protein revealed SP (-8.033 kcal/mol), XPG (-8.033 kcal/mol) and MM-GBSA (-63.85 kcal/mol) binding scores while rivastigmine with acetylcholinesterase showed SP (-8.248 kcal/mol), XPG (-8.297 kcal/mol) and MM-GBSA (-61.88 kcal/mol) binding scores as seen in Table S7. However, gamma-tocopherol with a MM-GBSA score of -63.85 kcal/mol indicates a binding affinity that favorably competes with the reference and much better than other compounds identified in PE*Hc*. As seen in Fig. 11Ab & Bb, hydrogen interaction of gamma-tocopherol with acetylcholinesterase revealed the formation of three hydrogen bonds at TYR124 (2.04097), TRP86 (2.77864), TRP86 (2.23506) whereas, interaction of rivastigmine with acetylcholinesterase protein formed two hydrogen bond at TYR124 (1.72327) and TYR341 (2.82678), as well as two electrostatic bonds at TRP286 (4.54556) and TRP286 (4.25351), respectively. However, the electrostatic bonds are precisely Pi-cation type of bond. Also, as shown in Fig. 11Ac & Bc, hydrophobic interaction of gamma-tocopherol with acetylcholinesterase protein formed hydrophobic bonds at TRP286 (2.87925), TRP86 (5.07953), TRP86 (5.44587), VAL294 (4.19175), LEU289 (4.96319), TYR72 (4.8553), TRP286 (4.75968), PHE297 (5.35238), TYR337 (4.8892), TYR337 (4.18453), PHE338 (5.02146), PHE338 (4.27883), PHE338 (5.27724), PHE338 (5.19853), TYR341 (5.27106), TYR341 (4.87417), TYR341 (4.18889), TYR341 (4.86564), HIS447 (4.83387), and HIS447 (4.50432), while eight hydrophobic bond are formed with rivastigmine at TRP286 (2.44916), TRP286 (2.86409), TRP286 (4.92554), TYR337 (3.88247), PHE338 (4.56608), PHE338 (4.64674), TYR341 (4.09141), and HIS447 (5.19716), respectively. However, the hydrophobic bonds formed two Pi-sigma at TRP286 (2.86409) and TRP286 (4.92554), five Pi-alkyl at TYR337 (3.88247), PHE338 (4.56608), PHE338 (4.64674), TYR341 (4.09141), and HIS447 (5.19716), and one Pi-Pi stacked at TRP286 (4.92554), respectively.

## Discussion

In recent years, there has been an increased interest and significant effort directed toward investigating target drugs capable of inhibiting both the pathophysiogenesis and progression of numerous human diseases, particularly those derived from natural plants [4, 66]. Polyphenolic compounds are commonly found in natural plants [67]. In this study (Fig. 1a, b & c), screening of PEHc showed antioxidative activity such as the bleaching of DPPH^•^ chromogenic radical to its corresponding hydrazine [2], inhibition of ABTS radical cation, which could be attributed to the available polyphenols (Fig. 1c and Table 2). These compounds constitute one of the most abundant groups of compounds that are important component of both human and animal nutrition [68], with verified antioxidative and non-antioxidative functions among which are ability to; interact with proteins and inhibit enzymatic activity, scavenge ROS or modulate pathways that regulate ROS scavenging systems, boost the immune system and cause termination of pathogenesis of disease conditions [2, 69, 70].

As seen in this study, PEHc showed substantial inhibitory activities against α-glucosidase and α-amylase enzymatic activities (Fig. 2a & b). The inhibition of enzymes involved in carbohydrate hydrolysis in the digestive organs has been reported as an important control measure of postprandial hyperglycemia in T2DM [13]. However, studies have associated polyphenols whose activities strongly been linked to their ability to donate H-atom (H^+^) as important agents in the inhibition of α-glucosidase and α-amylase [71], with redox potential of their hydroxyl groups being implicated as mechanisms involved in the inhibition [72]. The observation of this study perhaps, could indicate the antidiabetic drug-able potentials of PEHc as well as validating the report that indicates hypoglycaemic properties of plant-rich polyphenolic compounds, and effects similar to insulin in the utilization/uptake of serum glucose [2, 73].

Similarly, the importance of plant extracts have been reported in the treatment of AD [74]. So far, most of the active drugs currently approved for the treatment of AD are inhibitors of AChE and BChE that directly contributes to regulation and memory processes [75]. In AD, there is cholinergic impairment characterised by elevated activity of AChE, making it an appropriate target for AD medication development [25]. Moreover, the inhibition of AChE plays a key role not only enhancing cholinergic transmission in the brain, as well as reducing the formation and aggregation of amyloid beta peptide in AD [76]. As indicated in this report, the PEHc caused a remarkable inhibition of AChE and BChE (Fig. 3a & b). It could therefore, be suggested that PEHc might possess a neuroprotective potentials that are useful in the management of AD [77], having demonstrated inhibitory effect against these enzymes.

### Molecular docking

In validating the molecular basis of interaction and drug-like potentials of the prominent lead compounds from PEHc within the binding pose of the protein targets, SP, XPG and MM-GBSA docking were carried out via molecular docking. Moreover, molecular docking is critical in the development and design of novel drugs. It accurately predicts the experimental binding mode and affinity of a native molecule within the drug target’s binding site [78]. Nevertheless, there have already been a number of studies on the application of docking for the design of novel multi-target ligands [79, 80]. The use of in silico methods like chemo-informatics, molecular modeling, and artificial intelligence (AI) has grown significantly over the past few decades giving the recent advancements in computer technology and rapid increase of structural, chemical, and biological data available on an ever-increasing number of therapeutic targets [81, 82].

According to this study, among the twelve HPLC-identified compounds from PEHc (Table 2), hibiscetin was found to have the lowest MM-GBSA scores, in addition to its high SP and XPG scores (Tables S1 and S2), thus, making hibiscetin to bind more stronger with the target proteins (1RWQ and 1SMD) as seen in Figs. 5A & B and 6A & B, compared to the standards (dutogliptin and acarbose). However, the use of MM-GBSA has been reported to have a better correlation to binding affinity of docked complexes compared to docking and post-scoring of compounds [83, 84]. MM-GBSA has also received a lot of attention as an advanced computational way of analysing binding energy with better algorithms and solvation modes [85]. Consequently, this observation obviously depicts the potential for drug-likeliness and polypharmacological nature of hibiscetin. In a similar manner, gamma-tocopherol was found to have also demonstrated polypharmacological properties by simultaneously and effectively binding with a pool of protein targets such as 3C59, 7B04, and 4EY7 (Figs. 7A & B, 10A & B and 11A & B), respectively. The exhibited SP, XPG and MM-GBSA binding affinity scores when interacted with these protein targets (Tables S3, S6 and S7) distinctively support this observation. This report corroborates previous studies where docking for the design of novel multi-target ligands were applied [80, 86].

In contrast to the potential polypharmacological properties demonstrated by hibiscetin and gamma-tocopherol, cianidanol indicated a stronger affinity for 7KBJ (Fig. 8A & B), strongly supported by the exhibited binding affinity scores (Table S4), while kaempferol precisely demonstrated a stronger binding affinity for 6BHV (Fig. 9A & B, Table S5). All these observations could be due to the contribution of both hydrogen and hydrophobic bonds interactions [87]. The lower binding affinities, which favors strong interaction that were observed in multiple-target properties of gamma-tocopherol and hibiscetin could be due to their extensive hydrogen, hydrophobic, electrostatic and other forms of interactions. However, study shows that hydrogen bonding is directional and so significant in the field of biomolecular recognition [88]. Hydrogen bonds are important not only for mediating drug-receptor binding, but also for influencing physicochemical aspects of a molecule such as solubility, partitioning, distribution, and permeability, all of which are important in drug development [89]. Hydrogen bonds and lipophilic connections are known to be most important contributions to protein-ligand interactions.

Similarly, formation of hydrophobic contacts could be because of close proximity of non-polar amino acid side chains of the protein and lipophilic groups on the ligand [90]. Hydrophobic interactions are regarded as a driving force for conformational changes of the receptor upon ligand binding [91]. Lipophilic groups include aliphatic or aromatic hydrocarbon groups, as well as halogen substituents (e.g., chlorine) and a variety of heterocycles such as thiophene and furan [92]. All portions of a protein’s or ligand’s surface that cannot establish H-bonds or other polar interactions are considered lipophilic. Hydrophobic interactions, unlike hydrogen bonds, are not directional [93], and have been reported to contribute significantly to binding affinity of ligands with substantial lipophilic groups [94]. Furthermore, study have shown the role of hydrophobic interactions to binding affinity via induction of proximity that is proportional to the size of the lipophilic surface buried upon ligand binding and hence, limit accessibility to water [95]. According to a report of Klebe [96], escape of water molecules from the lipophilic environment of the binding pockets, could perhaps be the basis for the formation of strong hydrophobic interactions of the lead compounds with all the protein targets as observed in the study.

Also, parameters such as absorption, distribution, metabolism, excretion and toxicity (ADMET) of the lead compounds of PEHc were evaluated as seen in Table 3. First, Caco-2, which is a human colorectal adenocarcinoma cell line that is commonly used to study the permeability of drugs across the intestinal barrier [97]. As indicated in the results, only gamma-tocopherol and kaempferol revealed better Caco-2 permeability predicted scores of >-5.15 log cm/s [98], indicating that gamma-tocopherol and kaempferol are better permeants of Caco-2. Also, Pgp, a membrane-bound efflux transporter that is involved in the transport of drugs and other compounds out of cells, which can affect drug absorption and distribution in the body [99]. It has been shown that Pgp inhibitors and substrates are important parameters to consider in assessing drug solubility and bioavailability [100]. As revealed in our report, all the compounds revealed excellent values as potent inhibitors and substrates for Pgp. This probably suggest that all the lead compounds possess drug-like effects with high levels of solubility and bioavailability. Similarly, all the compounds revealed excellent scores for HIA, which is an essential prerequisite for drug apparent efficacy [101].

**Table 3:** ADMET properties of lead compounds from polyphenolic-rich extract of *Hibiscus cannabinus* (PEHc)

Compounds	Absorption				Distribution			Metabolism		Excretion	Toxicity		
	Caco-2-Permeability	Pgp-inhibitor	Pgp-substrate	HIA	PPB (%)	VD	BBB-Penetration	Inhibitor	Substrate	CL	hERG-Blockers	AMES Toxicity	Carcin
Cianidanol	-6.052	0.008	0.010	0.035	89.2	0.656	0.029	CYP1A2, CYP3A4, CYP2C19, CYP2C9, CYP2D6	CYP2C9, CYP1A2, CYP3A4, CYP2C19, CYP2D6	17.30	0.033	0.604	0.185
ϒ-Tocopherol	-4.991	0.040	0.001	0.002	100.7	4.565	0.916	CYP1A2, CYP2C19, CYP2C9, CYP2D6, CYP3A4	CYP1A2, CYP2C9, CYP2C19, CYP2D6, CYP3A4	6.64	0.023	0.037	0.029
Hibiscetin	-5.961	0.006	0.002	0.089	91.79	0.656	0.003	CYP1A2, CYP2C19, CYP2C9, CYP2D6, CYP3A4	CYP2C19, CYP1A2, CYP2C9, CYP2D6, CYP3A4	8.23	0.142	0.595	0.036
Kaempferol	-4.974	0.004	0.011	0.008	97.86	0.522	0.009	CYP1A2, CYP2C19, CYP2C9, CYP2D6, CYP3A4	CYP1A2, CYP2C19, CYP2C9, CYP2D6, CYP3A4	6.87	0.070	0.672	0.097

Key: *Caco-2* Human colon adenocarcinoma cell lines (log cm/s; permeability predicted value > -5.15 log cm/s)

*Pgp* P-glycoprotein; inhibitor or substrate (Probability; 0–0.3: excellent; 0.3–0.7 medium; 0.7–1.0(+ +): poor)

*HIA* Human intestinal absorption (Probability; 0–0.3: excellent; 0.3–0.7: medium; 0.7–1.0(+ +): poor)

*VD* Volume of distribution (log L/kg; excellent: 0.04–20; otherwise: poor)

*CYP* Cytochrome

*PPB* Plasma protein binding (Excellent ≤ 90%; otherwise- poor)

*BBB-Penetration* Blood–brain barrier penetration (Probability; excellent: 0–0.3; medium: 0.3–0.7; poor: 0.7–1.0(+ +)

*CL* Clearance (log ml/min/kg; excellent: ≥ 5; poor: < 5)

hERG-Blockers (Excellent: 0–0.3; medium: 0.3–0.7; poor: 0.7–1.0(+ +))

AMES Toxicity (Excellent: 0–0.3; medium: 0.3–0.7; poor: 0.7–1.0(+ +))

*Carcin* Carcinogencity (Excellent: 0–0.3; medium: 0.3–0.7; poor: 0.7–1.0(+ +))

During drug development and evaluation, drug distribution is critical, and several factors such as PPB, Vd, and BBB penetration are important considerations [102]. These parameters can impact the efficacy of drugs by affecting their distribution to target tissues and organs. As shown in this study (Table 3), only cianidanol had excellent PPB score while the remaining compounds had values exceeding > 90 PPB scores. Study has shown that highly protein-bound drugs are less likely to diffuse across cell membranes and are therefore less available for distribution to tissues, whereas drugs that are not protein-bound have a higher degree of distribution and therapeutic index [103]. Also, as shown in the results (Table 3), all the lead compounds had excellent Vd scores, i.e., between 0.04–20, which could probably be connected to their physicochemical properties. More so, BBB values of the lead compounds were evaluated. The BBB is a specialized structure that prevents many drugs from entering the brain, limiting their efficacy in treating neurological disorders. Drugs that are able to penetrate the BBB have a higher degree of distribution within the brain and are more likely to be effective in treating neurological disorders [104].

The metabolism and total clearance are important parameters when evaluating drug pharmacokinetic [105]. As shown in the results, all the lead compounds from PEHc revealed good pharmacokinetic-related values that are useful in drug design. More so, assessment of hERG- blockers, AMES toxicity and carcinogenicity for possible toxicity were performed. All the lead compounds showed drug-likeness values for hERG-blockers with an indication that they are not carcinogenic. However, among the lead compounds only gamma-tocopherol had an excellent AMES toxicity value. This suggests that gamma-tocopherol might not be toxic and could be a drug-able compound.

## Conclusion

In this study, the polyphenolic-rich extract of *Hibiscus cannabinus* seed demonstrated noticeable antioxidant potentials with a significant inhibition of some enzymes that are implicated in type II diabetes mellitus (DM) and Alzheimer’s disease (AD), an observation that could be credited to its HPLC-identified bioactive compounds. Similarly, in silico docking and computational prediction of HPLC-identified compounds indicated prospective drug-like interactions of hibiscetin, gamma-tocopherol and cianidanol with some protein targets in DM and AD. Consequently, the findings of this study suggest the prospective drug-able compounds in polyphenolic-rich extract of *Hibiscus cannabinus* seed that might be useful in the management of DM and AD.

## Supplementary Information

**Additional file 1: Table S1.** Post docking SP, XPG and MMGBSA scores of compounds from polyphenolic-rich extract of *Hibiscus cannabinus* and a standard (dutogliptin) with dipeptidyl peptidase IV (1RWQ). **Table S2.** Post docking SP, XPG and MM-GBSA scores of compounds from polyphenolic-rich extract of *Hibiscus cannabinus* and a standard (acarbose) with alpha amylase (1SMD). **Table S3.** Post docking SP, XPG and MM-GBSA scores of compounds from polyphenolic-rich extract of *Hibiscus cannabinus* and a standard (Cochinchinenin C) with glucagon-like peptide-1 receptor (3C59). **Table S4.** Post docking SP, XPG and MM-GBSA scores of compounds from polyphenolic-rich extract of *Hibiscus cannabinus* and a standard (Acarbose) with alpha glucosidase (7KBJ). **Table S5.** Post docking SP, XPG and MM-GBSA scores of compounds from polyphenolic-rich extract of *Hibiscus cannabinus* and a standard (3-aminobenzamide) with poly [ADP-ribose] polymerase 1 (6BHV). **Table S6.** Post docking SP, XPG and MM-GBSA scores of compounds from polyphenolic-rich extract of *Hibiscus cannabinus* and a standard (Rivastigmine) with Butylcholinesterase (7B04). **Table S7.** Post docking SP, XPG and MM-GBSA scores of compounds from polyphenolic-rich extract of *Hibiscus cannabinus* and a standard (Rivastigmine) with Acetylcholinestrase (4EY7).

## Data Availability

Proteins analyzed such as PDB ID: 1RWQ, PDB ID: 1SMD, PDB ID: 3C59, PDB ID: 4EY7, PDB ID: 7B04, PDB ID: 7KBJ and PDB ID: 6BHV were obtained from protein data bank (https://www.rcsb.org/).

## References

[1] Zhang W, Bai M, Xi Y, Hao J, Liu L, Mao N (2012). Early memory deficits precede plaque deposition in APPswe/PS1dE9 mice: involvement of oxidative stress and cholinergic dysfunction. Free Rad Biol Med.

[2] Afolabi OB, Oloyede OI, Agunbiade SO (2018). Inhibitory potentials of phenolic-rich extracts from *Bridelia ferruginea* on two key carbohydrate-metabolizing enzymes and Fe^2+^-induced pancreatic oxidative stress. J Integr Med.

[3] Pasquier F, Boulogne A, Leys D, Fontaine P (2006). Diabetes mellitus and dementia. Diabetes Metab.

[4] Ogunsanmi AO, Raheem T, Adio WS (2022). Molecular aspect of diabetes mellitus. World Sci News.

[5] Ezhilarasan D (2018). Oxidative stress is bane in chronic liver diseases: Clinical and experimental perspective. Arab J Gastroenterol.

[6] Kruk J, Aboul-Enein HY, Kładna A, Bowser JE (2019). Oxidative stress in biological systems and its relation with pathophysiological functions: the effect of physical activity on cellular redox homeostasis. Free Rad Res.

[7] Marrero DG, Ard J, Delamater AM, Peragallo-Dittko V, Mayer-Davis EJ, Nwankwo R (2013). Twenty-first century behavioral medicine: a context for empowering clinicians and patients with diabetes: a consensus report. Diabetes Care.

[8] Briançon-Marjollet A, Weiszenstein M, Henri M, Thomas A, Godin-Ribuot D, Polak J (2015). The impact of sleep disorders on glucose metabolism: endocrine and molecular mechanisms. Diabetol Metab Syndr.

[9] Roglic G (2016). WHO global report on diabetes: a summary. Int J Noncomm Dis.

[10] Moore PA, Zgibor JC, Dasanayake AP (2003). Diabetes: a growing epidemic of all ages. J Am Dent Ass.

[11] Katakami N (2018). Mechanism of development of atherosclerosis and cardiovascular disease in diabetes mellitus. J Atheroscler Thromb.

[12] Esmaeili S, Azizian S, Shahmoradi B, Moradi S, Shahlaei M, Khodarahmi R (2019). Dipyridamole inhibits α-amylase/α-glucosidase at sub-micromolar concentrations; *in-vitro*, *in-vivo* and theoretical studies. Bioorg Chem.

[13] Afolabi OB, Oloyede OI, Aluko BT, Johnson JA (2021). Biosynthesis of magnesium hydroxide nanomaterials using *Monodora myristica*, antioxidative activities and effect on disrupted glucose metabolism in streptozotocin-induced diabetic rat. Food Biosci.

[14] Xu G, Liu B, Sun Y, Du Y, Snetselaar LG, Hu FB (2018). Prevalence of diagnosed type 1 and type 2 diabetes among US adults in 2016 and 2017: population based study. BMJ.

[15] Xu H, Du X, Xu J, Zhang Y, Tian Y, Liu G (2020). Pancreatic β cell microRNA-26a alleviates type 2 diabetes by improving peripheral insulin sensitivity and preserving β cell function. PLoS Biol.

[16] Brown OI, Bridge KI, Kearney MT (2021). Nicotinamide adenine dinucleotide phosphate oxidases in glucose homeostasis and diabetes-related endothelial cell dysfunction. Cells.

[17] Porte D, Kahn SE (2001). beta-cell dysfunction and failure in type 2 diabetes: potential mechanisms. Diabetes.

[18] Smith DL, Orlandella RM, Allison DB, Norian LA (2021). Diabetes medications as potential calorie restriction mimetics—a focus on the alpha-glucosidase inhibitor acarbose. GeroScience.

[19] Williamson G (2013). Possible effects of dietary polyphenols on sugar absorption and digestion. Mol Nutr Food Res.

[20] Desai NR, Shrank WH, Fischer MA, Avorn J, Liberman JN, Schneeweiss S (2012). Patterns of medication initiation in newly diagnosed diabetes mellitus: quality and cost implications. Am J Med.

[21] Duffy NA, Green BD, Irwin N, Gault VA, McKillop AM, O’Harte FP (2007). Effects of antidiabetic drugs on dipeptidyl peptidase IV activity: nateglinide is an inhibitor of DPP IV and augments the antidiabetic activity of glucagon-like peptide-1. Eur J Pharmacol.

[22] Xue M, Xu W, Ou YN, Cao XP, Tan MS, Tan L (2019). Diabetes mellitus and risks of cognitive impairment and dementia: a systematic review and meta-analysis of 144 prospective studies. Ageing Res Rev.

[23] He JT, Zhao X, Xu L, Mao CY (2020). Vascular risk factors and Alzheimer’s disease: blood-brain barrier disruption, metabolic syndromes, and molecular links. J Alzheimers Dis.

[24] Gupta R, Sen N (2016). Traumatic brain injury: a risk factor for neurodegenerative diseases. Rev Neurosci.

[25] Kumar A, Pintus F, Di Petrillo A, Medda R, Caria P, Matos MJ (2018). Novel 2-pheynlbenzofuran derivatives as selective butyrylcholinesterase inhibitors for Alzheimer’s disease. Sci Rep.

[26] Akanji MA, Rotimi DE, Elebiyo TC, Awakan OJ, Adeyemi OS (2021). Redox homeostasis and prospects for therapeutic targeting in neurodegenerative disorders. Oxid Med Cell Longev.

[27] Massaad CA, Klann E (2011). Reactive oxygen species in the regulation of synaptic plasticity and memory. Antioxid Redox Signal.

[28] Gao H, Jiang Y, Zhan J, Sun Y (2021). Pharmacophore-based drug design of AChE and BChE dual inhibitors as potential anti-Alzheimer’s disease agents. Bioorg Chem.

[29] Silman I (2021). The multiple biological roles of the cholinesterases. Prog Biophys Mol Biol.

[30] Ha ZY, Mathew S, Yeong KY (2020). Butyrylcholinesterase: a multifaceted pharmacological target and tool. Curr Protein Pept Sci.

[31] Ali MY, Jannat S, Edraki N, Das S, Chang WK, Kim HC (2019). Flavanone glycosides inhibit β-site amyloid precursor protein cleaving enzyme 1 and cholinesterase and reduce Aβ aggregation in the amyloidogenic pathway. Chem Biol Interact.

[32] Marucci G, Buccioni M, Dal BD, Lambertucci C, Volpini R, Amenta F (2021). Efficacy of acetylcholinesterase inhibitors in Alzheimer’s disease. Neuropharmacology.

[33] Schneider LJ (2001). Treatment of Alzheimer’s disease with cholinesterase inhibitors. Clin Geriatr Med.

[34] Carrasco-Ramiro F, Peiró-Pastor R, Aguado B (2017). Human genomics projects and precision medicine. Gene Ther.

[35] Ain QU, Aleksandrova A, Roessler FD, Ballester PJ (2015). Machine-learning scoring functions to improve structure-based binding affinity prediction and virtual screening. Wiley Interdiscip Rev Comput Mol Sci.

[36] Zoete V, Schuepbach T, Bovigny C, Chaskar P, Daina A, Röhrig UF (2016). Attracting cavities for docking. Replacing the rough energy landscape of the protein by a smooth attracting landscape. J Comput Chem..

[37] Pantsar T, Poso A (2018). Binding affinity via docking: fact and fiction. Molecules.

[38] Sliwoski G, Kothiwale S, Meiler J, Lowe EW (2014). Computational methods in drug discovery. Pharmacol Rev.

[39] Nguyen DD, Wei GW (2019). AGL-score: algebraic graph learning score for protein–ligand binding scoring, ranking, docking, and screening. J Chem Inf Model.

[40] Gandhi NS, Mancera RL (2008). The structure of glycosaminoglycans and their interactions with proteins. Chem Biol Drug Des.

[41] Bhatt S, Puli L, Patil CR (2021). Role of reactive oxygen species in the progression of Alzheimer’s disease. Drug Discov Today.

[42] Cheng Z, Lu BR, Sameshima K, Fu DX, Chen JK (2004). Identification and genetic relationships of kenaf (*Hibiscus cannabinus* L.) germplasm revealed by AFLP analysis. Genet Resour Crop Evol.

[43] Ayadi R, Hanana M, Mzid R, Hamrouni L, Khouja ML, Salhi Hanachi A (2017). *Hibiscus cannabinus* L.–kenaf: a review paper. J Nat Fibers.

[44] Afzal MZ, Ibrahim AK, Xu Y, Niyitanga S, Li Y, Li D (2022). Kenaf (*Hibiscus cannabinus* L.) breeding. J Nat Fibers.

[45] Ahmed F, Das A, Sarker S, Hasan MM, Ahmed SU (2012). Phytochemical screening and evaluation of antioxidant and antimicrobial activities of *Hibiscus cannabinus* Linn. J Appl Pharm Sci.

[46] Gupta A, Sharma T, Singh SP, Bhardwaj A, Srivastava D, Kumar R. Prospects of microgreens as budding living functional food: Breeding and biofortification through OMICS and other approaches for nutritional security. Frontiers in Genet. 2023;14. 10.3389/fgene.2023.1053810 PMC9905132 36760994

[47] Viado AE, Purnamasari L, dela Cruz JF.  (2022). Anti-diabetic effects of Hibiscus spp. extract in rat and mice models: a review. Indonesian J Nutr.

[48] Chu Y, Sun J, Wu X, Liu RH (2002). Antioxidant and antiproliferative activity of common vegetables. J Agric Food Chem.

[49] Singleton VL, Orthofer R, Lamuela-Raventós RM (1999). [14] Analysis of total phenols and other oxidation substrates and antioxidants by means of folin-ciocalteu reagent. Methods Enzymol.

[50] Bao J, Cai Y, Sun M, Wang G, Corke H (2005). Anthocyanins, flavonols and free radical scavenging activity of Chinese bayberry (Myrica rubra) extracts and their color properties and stability. J Agric Food Chem.

[51] Gyamfi MA, Yonamine M, Aniya Y (1999). Free-radical scavenging action of medicinal herbs from Ghana: *Thonningia sanguinea* on experimentally-induced liver injuries. Gen Pharmacol Vascul Syst.

[52] Zhao H, Dong J, Lu J, Chen J, Li Y, Shan L (2006). Effects of extraction solvent mixtures on antioxidant activity evaluation and their extraction capacity and selectivity for free phenolic compounds in barley (*Hordeum vulgare* L.). J Agric Food Chem.

[53] Shai LJ, Masoko P, Mokgotho MP, Magano SR, Mogale AM, Boaduo N (2010). Yeast alpha-glucosidase inhibitory and antioxidant activities of six medicinal plants collected in Phalaborwa, South Africa. S Afr J Bot.

[54] Ademiluyi A, Oboh G (2013). Soybean phenolic-rich extracts inhibit key-enzymes linked to type 2 diabetes (α-amylase and α -glucosidase) and hypertension (angiotensin I converting enzyme) *in vitro*. Exp Toxicol Pathol.

[55] Perry NS, Houghton PJ, Theobald A, Jenner P, Perry EK (2000). *In vitro* activity of *S. lavandulaefolia* (Spanish sage) relevant to treatment of Alzheimer’s disease. J Pharm Pharmacol.

[56] Olasehinde OR, Afolabi OB, Omiyale BO, Olaoye OA (2021). *In vitro* inhibitory potentials of ethanolic extract of *Moringa oleifera* flower against enzymes activities linked to diabetes. J Herbmed Pharmacol.

[57] Afolabi OB, Oloyede OI, Agunbiade OS, Obafemi TO, Aline B, Obajuluwa A (2019). HPLC-DAD profiling and inhibitory potentials of ethylacetate and aqueous extracts of *Talinum triangulare* on key enzymes linked to type-2 diabetes (α-amylase and α-glucosidase) and oxidative stress (monoamine oxidase). Egypt J Basic Appl Sci.

[58] Repasky MP, Shelley M, Friesner RA (2007). Flexible ligand docking with Glide. Curr Protoc Bioinformatics.

[59] Mahmoud DE, Faraag AH, Abu El-Wafa WM (2021). *In vitro* study on the potential fungicidal effects of atorvastatin in combination with some azole drugs against multidrug resistant *Candida albicans*. World J Microbiol Biotechnol.

[60] Tripathi SK, Muttineni R, Singh SK (2013). Extra precision docking, free energy calculation and molecular dynamics simulation studies of CDK2 inhibitors. J Theor Biol.

[61] Halgren TA, Murphy RB, Friesner RA, Beard HS, Frye LL, Pollard WT (2004). Glide: a new approach for rapid, accurate docking and scoring. 2. Enrichment factors in database screening. J Med Chem.

[62] Friesner RA, Banks JL, Murphy RB, Halgren TA, Klicic JJ, Mainz DT (2004). Glide: a new approach for rapid, accurate docking and scoring. 1. Method and assessment of docking accuracy. J Med Chem.

[63] Das D, Koh Y, Tojo Y, Ghosh AK, Mitsuya H (2009). Prediction of potency of protease inhibitors using free energy simulations with polarizable quantum mechanics-based ligand charges and a hybrid water model. J Chem Inf Model.

[64] Du J, Sun H, Xi L, Li J, Yang Y, Liu H (2011). Molecular modeling study of checkpoint kinase 1 inhibitors by multiple docking strategies and prime/MM–GBSA calculation. J Comput Chem.

[65] Lyne PD, Lamb ML, Saeh JC (2006). Accurate prediction of the relative potencies of members of a series of kinase inhibitors using molecular docking and MM-GBSA scoring. J Med Chem.

[66] Loizzo MR, Tundis R, Menichini F, Menichini F (2008). Natural products and their derivatives as cholinesterase inhibitors in the treatment of neurodegenerative disorders: an update. Curr Med Chem.

[67] Ohri P, Pannu SK (2010). Effect of phenolic compounds on nematodes–a review. J Appl Nat Sci.

[68] Valdés L, Cuervo A, Salazar N, Ruas-Madiedo P, Gueimonde M, González S (2015). The relationship between phenolic compounds from diet and microbiota: impact on human health. Food Funct.

[69] Hoffmann A, Kleniewska P, Pawliczak R (2021). Antioxidative activity of probiotics. Arc Med Sci.

[70] Huang WY, Cai YZ, Zhang Y (2009). Natural phenolic compounds from medicinal herbs and dietary plants: potential use for cancer prevention. Nutr Cancer.

[71] Kalita D, Holm DG, LaBarbera DV, Petrash JM, Jayanty SS (2018). Inhibition of α-glucosidase, α-amylase, and aldose reductase by potato polyphenolic compounds. PLoS One.

[72] Ali Asgar MD (2013). Anti-diabetic potential of phenolic compounds: a review. Int J Food Prop.

[73] Mahmood N (2016). A review of α-amylase inhibitors on weight loss and glycemic control in pathological state such as obesity and diabetes. Comp Clin Pathol.

[74] Orhan I, Şener B, Choudhary MI, Khalid A (2004). Acetylcholinesterase and butyrylcholinesterase inhibitory activity of some Turkish medicinal plants. J Ethnopharmacol.

[75] Konrath EL, Passos CD, Klein-Júnior LC, Henriques AT (2013). Alkaloids as a source of potential anticholinesterase inhibitors for the treatment of Alzheimer’s disease. J Pharm Pharmacol.

[76] Ibrahim MM, Gabr MT (2019). Multitarget therapeutic strategies for Alzheimer’s disease. Neural Regen Res.

[77] Lovinger DM (2010). Neurotransmitter roles in synaptic modulation, plasticity and learning in the dorsal striatum. Neuropharmacology.

[78] Pinzi L, Rastelli G (2019). Molecular docking: shifting paradigms in drug discovery. Int J Mol Sci.

[79] Lepailleur A, Freret T, Lemaître S, Boulouard M, Dauphin F, Hinschberger A (2014). Dual histamine H3R/serotonin 5-HT4R ligands with antiamnesic properties: pharmacophore-based virtual screening and polypharmacology. J Chem Inf Model.

[80] Anighoro A, Bajorath J (2017). Compound ranking based on fuzzy three-dimensional similarity improves the performance of docking into homology models of g-protein-coupled receptors. ACS Omega.

[81] Jorgensen WL (2004). The many roles of computation in drug discovery. Science.

[82] Macalino SJ, Gosu V, Hong S, Choi S (2015). Role of computer-aided drug design in modern drug discovery. Arch Pharm Res.

[83] Mali SN, Chaudhari HK (2019). Molecular modelling studies on adamantane-based Ebola virus GP-1 inhibitors using docking, pharmacophore and 3D-QSAR. SAR QSAR Environ Res.

[84] Pattar SV, Adhoni SA, Kamanavalli CM, Kumbar SS (2020). *In silico* molecular docking studies and MM/GBSA analysis of coumarin-carbonodithioate hybrid derivatives divulge the anticancer potential against breast cancer. Beni-Suef Univ J Basic App Sci.

[85] Greenidge PA, Kramer C, Mozziconacci JC, Wolf RM (2013). MM/GBSA binding energy prediction on the PDBbind data set: successes, failures, and directions for further improvement. J Chem Inf Model.

[86] Lan JS, Ding Y, Liu Y, Kang P, Hou JW, Zhang XY (2017). Design, synthesis and biological evaluation of novel coumarin-N-benzyl pyridinium hybrids as multi-target agents for the treatment of Alzheimer’s disease. Eur J Med Chem.

[87] Dinh NP, Jonsson T, Irgum K (2011). Probing the interaction mode in hydrophilic interaction chromatography. J Chromatogr A.

[88] Coulocheri SA, Pigis DG, Papavassiliou KA, Papavassiliou AG (2007). Hydrogen bonds in protein–DNA complexes: where geometry meets plasticity. Biochimie.

[89] Hopkins AL, Keserü GM, Leeson PD, Rees DC, Reynolds CH (2014). The role of ligand efficiency metrics in drug discovery. Nat Rev Drug Discov.

[90] Yaacob N, Ali MS, Salleh AB, Rahman RN, Leow AT (2016). Toluene promotes lid 2 interfacial activation of cold active solvent tolerant lipase from Pseudomonas fluorescens strain AMS8. J Mol Graph Model.

[91] Zhang N, Cui Z, Li M, Fan Y, Liu J, Wang W (2022). Typical Umami ligand-induced binding interaction and conformational change of T1R1-VFT. J Agric Food Chem.

[92] Guglielmi P, Mathew B, Secci D, Carradori S (2020). Chalcones: unearthing their therapeutic possibility as monoamine oxidase B inhibitors. Eur J Med Chem.

[93] Sikder A, Ghosh S (2019). Hydrogen-bonding regulated assembly of molecular and macromolecular amphiphiles. Mat Chem Front.

[94] Perola E (2010). An analysis of the binding efficiencies of drugs and their leads in successful drug discovery programs. J Med Chem.

[95] Wasan KM, Brocks DR, Lee SD, Sachs-Barrable K, Thornton SJ (2008). Impact of lipoproteins on the biological activity and disposition of hydrophobic drugs: implications for drug discovery. Nat Rev Drug Discov.

[96] Klebe G (2015). Applying thermodynamic profiling in lead finding and optimization. Nat Rev Drug Discov.

[97] Artursson P, Karlsson J (1991). Correlation between oral drug absorption in humans and apparent drug permeability coefficients in human intestinal epithelial (Caco-2) cells. Biochem Biophys Res Comm.

[98] Castillo-Garit JA, Marrero-Ponce Y, Torrens F, García-Domenech R. Estimation of ADME properties in drug discovery: predicting Caco-2 cell permeability using atom-based stochastic and non-stochastic linear indices. J Pharm Sci. 2008;97(5):1946–76. 10.1002/jps.21122 17724669

[99] Zhang L, Zhang Y (2018). P-glycoprotein/ABCB1 in cancer: learn from pharmacogenomic research. Front Pharmacol.

[100] Wessler JD, Grip LT, Mendell J (2016). Pharmacokinetics and pharmacodynamics of P-glycoprotein modulators. Clin Pharm.

[101] Pantaleão SQ, Fernandes PO, Gonçalves JE, Maltarollo VG, Honorio KM (2022). Recent advances in the prediction of pharmacokinetics properties in drug design studies: a review. Chem Med Chem.

[102] Zhou W, Wang Y, Lu A, Zhang G (2016). Systems pharmacology in small molecular drug discovery. Int J Mol Sci.

[103] Benet LZ, Hoener BA (2002). Changes in plasma protein binding have little clinical relevance. Clin Pharmacol Ther.

[104] Pardridge WM (2012). Drug transport across the blood-brain barrier. J Cerebral Blood Flow Metab.

[105] Kazakova O, Lopatina T, Giniyatullina GN, Mioc M, Soica C (2020). Antimycobacterial activity of azepanobetulin and its derivative: *In vitro*, *in vivo*, ADMET and docking studies. Bioorg Chem.

