# Supplementary material for: Antioxidant evaluation and computational prediction of prospective drug-like compounds from polyphenolic-rich extract of Hibiscus cannabinus L. seed as antidiabetic and neuroprotective targets: assessment through in vitro and in silico studies
Source: BMC Complement Med Ther. 2023 Jun 19;23:203. doi: 10.1186/s12906-023-04023-7 (PMC10280950; doi:10.1186/s12906-023-04023-7)
Supplement: Supplementary file 1 — Additional file 1: Table S1. Post docking SP, XPG and MMGBSA scores of compounds from polyphenolic-rich extract of Hibiscus cannabinus and a standard (dutogliptin) with dipeptidyl peptidase IV (1RWQ). Table S2. Post docking SP, XPG and MM-GBSA scores of compounds from polyphenolic-rich extract of Hibiscus cannabinus and a standard (acarbose) with alpha amylase (1SMD). Table S3. Post docking SP, XPG and MM-GBSA scores of compounds from polyphenolic-rich extract of Hibiscus cannabinus and a standard (Cochinchinenin C) with glucagon-like peptide-1 receptor (3C59). Table S4. Post docking SP, XPG and MM-GBSA scores of compounds from polyphenolic-rich extract of Hibiscus cannabinus and a standard (Acarbose) with alpha glucosidase (7KBJ). Table S5. Post docking SP, XPG and MM-GBSA scores of compounds from polyphenolic-rich extract of Hibiscus cannabinus and a standard (3-aminobenzamide) with poly [ADP-ribose] polymerase 1 (6BHV). Table S6. Post docking SP, XPG and MM-GBSA scores of compounds from polyphenolic-rich extract of Hibiscus cannabinus and a standard (Rivastigmine) with Butylcholinesterase (7B04). Table S7. Post docking SP, XPG and MM-GBSA scores of compounds from polyphenolic-rich extract of Hibiscus cannabinus and a standard (Rivastigmine) with Acetylcholinestrase (4EY7). [file 12906_2023_4023_MOESM1_ESM.docx]

Table S1: Post docking SP, XPG and MMGBSA scores of compounds from polyphenolic-rich extract of *Hibiscus cannabinus* and a standard (dutogliptin) with dipeptidyl peptidase IV (1RWQ).

| S/N | 1RWQ [Dipeptidyl peptidase IV] | | | |
| --- | --- | --- | --- | --- |
|  | PubChem ID | SP score | XPG score | MM-GBSA score |
| 1. | Hibiscetin | -7.522 | -7.559 | -40.22 |
| 2. | Cianidanol | -5.441 | -5.441 | -27.51 |
| 3. | Kaempferol | -5.182 | -5.214 | -27.03 |
| 4. | Beta-Sitosterol | -2.753 | -2.753 | -40.34 |
| 5. | Caffeic acid | -3.759 | -3.759 | -17.91 |
| 6. | Dutogliptin | -9.342 | -9.351 | -35.04 |

Table S2: Post docking SP, XPG and MM-GBSA scores of compounds from polyphenolic-rich extract of *Hibiscus cannabinus* and a standard (acarbose) with alpha amylase (1SMD).

| S/N | 1SMD [Alpha amylase] | | | |
| --- | --- | --- | --- | --- |
|  | PubChem ID | SP score | XPG score | MM-GBSA score |
| 1. | Hibiscetin | -9.303 | -9.34 | -35.25 |
| 2. | Cianidanol | -7.564 | -7.564 | -33.97 |
| 3. | Kaempferol | -7.345 | -7.377 | -33.54 |
| 4. | Beta-Sitosterol | -5.061 | -5.061 | -41.95 |
| 5. | Acarbose | -12.011 | -12.339 | -78.1 |

Table S3: Post docking SP, XPG and MM-GBSA scores of compounds from polyphenolic-rich extract of *Hibiscus cannabinus* and a standard (Cochinchinenin C) with glucagon-like peptide-1 receptor (3C59).

| S/N | 3C59 [Glucagon-like peptide-1 receptor] | | | |
| --- | --- | --- | --- | --- |
|  | PubChem ID | SP score | XPG score | MM-GBSA score |
| 1. | gamma-Tocopherol | -3.137 | -3.137 | -46.36 |
| 2. | beta-Sitosterol | -1.959 | -1.959 | -40.37 |
| 3. | Kaempferol | -2.945 | -2.977 | -36.74 |
| 4. | Hibiscetin | -3.800 | -3.837 | -34.74 |
| 5. | Ferulic acid | -2.318 | -2.318 | -26.30 |
| 6. | Cochinchinenin C | -2.631 | -2.637 | -53.43 |

Table S4: Post docking SP, XPG and MM-GBSA scores of compounds from polyphenolic-rich extract of *Hibiscus cannabinus* and a standard (Acarbose) with alpha glucosidase (7KBJ).

| S/N | 7KBJ [Alpha glucosidase] | | | |
| --- | --- | --- | --- | --- |
|  | PubChem ID | SP score | XPG score | MM-GBSA score |
| 1. | Hibiscetin | -8.126 | -8.163 | -27.79 |
| 2. | Cianidanol | -7.486 | -7.486 | -33.67 |
| 3. | Kaempferol | -4.6 | -4.6 | -2.15 |
| 4. | Beta-Sitosterol | -5.288 | -5.288 | -51.51 |
| 5. | Acarbose | -11.068 | -11.396 | -51.51 |

Table S5: Post docking SP, XPG and MM-GBSA scores of compounds from polyphenolic-rich extract of *Hibiscus cannabinus* and a standard (3-aminobenzamide) with poly [ADP-ribose] polymerase 1 (6BHV).

| S/N | 6BHV [Poly [ADP-ribose] polymerase 1] | | | |
| --- | --- | --- | --- | --- |
|  | PubChem ID | SP score | XPG score | MM-GBSA score |
| 1. | kaempferol | -9.046 | -9.078 | -61.69 |
| 2. | Hibiscetin | -10.771 | -10.808 | -58.97 |
| 3. | Cianidanol | -8.836 | -8.836 | -50.35 |
| 4. | gamma-Tocopherol | -6.222 | -6.222 | -56.15 |
| 5. | beta-Sitosterol | -5.004 | -5.004 | -47.74 |
| 6. | 3-Aminobenzamide | -6.646 | -6.646 | -35.39 |

Table S6: Post docking SP, XPG and MM-GBSA scores of compounds from polyphenolic-rich extract of *Hibiscus cannabinus* and a standard (Rivastigmine) with Butylcholinesterase (7B04).

| S/N | 7B04 [Butylcholinesterase] | | | |
| --- | --- | --- | --- | --- |
|  | PubChem ID | SP score | XPG score | MM-GBSA score |
| 1. | gamma-Tocopherol | -7.716 | -7. 716 | -49.97 |
| 2. | Kaempferol | -9.467 | -9.499 | -48.18 |
| 3. | Cianidanol | -8.807 | -8.807 | -45.99 |
| 4. | Kaempferol | -7.122 | -8.989 | -32.41 |
| 5. | Linalool | -3.773 | -3.773 | -19.91 |
| 6. | Rivastigmine | -6.644 | -6.693 | -49.00 |

Table S7: Post docking SP, XPG and MM-GBSA scores of compounds from polyphenolic-rich extract of *Hibiscus cannabinus* and a standard (Rivastigmine) with Acetylcholinestrase (4EY7).

| S/N | 4EY7 [Acetylcholinesterase] | | | |
| --- | --- | --- | --- | --- |
|  | PubChem ID | SP score | XPG score | MM-GBSA score |
| 1. | gamma-Tocopherol | -8.033 | -8.033 | -63.85 |
| 2. | Cianidanol | -10.741 | -10.741 | -61.20 |
| 3. | Kaempferol | -11.051 | -11.051 | -49.27 |
| 4. | Hibiscetin | -11.345 | -11.382 | -43.39 |
| 5. | Linalool | -4.868 | -4.868 | -35.12 |
| 6. | Rivastigmine | -8.248 | -8.297 | -61.88 |
